# Advance in the assembly of the plant mitochondrial genomes using high‐throughput DNA sequencing data of total cellular DNAs

**DOI:** 10.1111/pbi.70249

**Published:** 2025-07-29

**Authors:** Yang Ni, Jingling Li, Yihui Tan, Guoan Shen, Chang Liu

**Affiliations:** ^1^ Center for Bioinformatics, State Key Laboratory for Quality Ensurance and Sustainable Use of Dao‐di Herbs, Institute of Medicinal Plant Development Chinese Academy of Medical Sciences, Peking Union Medical College Beijing China

**Keywords:** plant mitochondrial genome, plant mitochondrial genome assembly algorithms, plant mitochondrial genome assembly tools, next‐generation DNA sequencing, third‐generation DNA sequencing

## Abstract

The assembly of plant mitochondrial genomes presents unique challenges due to difficulties in isolating mitochondrial DNA (mtDNA) and plant mitochondrial genome characteristics, such as low interspecific conservation; sequence sharing among mitochondrial, nuclear and plastid DNAs; and complex structural variations. Our laboratory has sequenced and assembled a dozen plant mitochondrial genomes, testing various strategies and identifying numerous assembly issues. This review compared the advanced methods and tools for plant mitochondrial genome assembly, categorizing assembly algorithms into three groups: (1) reference‐based, (2) de novo and (3) iterative mapping and extension. The performance of 11 software tools used most frequently over the past 5 years (GetOrganelle, Velvet, NOVOPlasty, SOAPdenovo2, Canu, Flye, SMARTdenovo, PMAT, NextDenovo, SPAdes and Unicycler) and two newly developed tools (TIPPo and Oatk) was assessed. The evaluation metrics included the completeness, contiguity and correctness of the assembled plant mitochondrial genomes. SMARTdenovo, NextDenovo and Oatk demonstrated superior performance in terms of contiguity and completeness. GetOrganelle and Flye excelled in correctness. Key challenges in plant mitochondrial genome assembly, such as removing nuclear mitochondrial DNA (NUMT) and mitochondrial plastid DNA (NUPT) contamination and resolving intra‐genomic repetitive regions, were discussed. A general strategy for plant mitochondrial genome assembly used in studies conducted in our laboratory was summarized. This review serves as a resource for those assembling plant mitochondrial genomes or developing plant mitochondrial genome assembly tools.

## Overview

The mitochondria are membrane‐bound organelles found in the cells of most eukaryotic organisms. Their primary function was to generate cellular adenosine triphosphate (ATP), the cell's primary energy currency (Harrington *et al*., 2023), through oxidative phosphorylation (Ghifari *et al*., 2023). Similar to plastids, the mitochondria have their own genomes. Elucidating the mitochondrial genome could help understand the mitochondria's evolutionary history, characterize the functions of proteins encoded by mitochondrial genomes and allow for manipulating mitochondrial functions through genome engineering.

In the past few years, our group has conducted several studies on plant mtDNA (Note S1). The results showed that plant mitochondrial genome sequencing has several challenges compared with that of nuclear DNAs (nuDNAs) and plastid DNAs (ptDNAs). First, plant cells have thicker cell walls and more organelles, which can interfere with the process of isolating complete plant mitochondria. The size, density and morphology of plant mitochondria vary among different species, requiring the optimization of the process to isolate the mitochondria of different species. Until now, the mitochondria have been successfully isolated and sequenced from species in a few plant lineages only, including *Arabidopsis thaliana* and various citrus species such as satsuma mandarin, Ponkan mandarin, sweet orange and pummelo (Li *et al*., 2021; Møller *et al*., 2021; Sweetlove *et al*., 2007).

As a result, plant mitochondrial genomes are mostly assembled from high‐throughput sequencing data generated from the total DNAs. The copy numbers of mtDNAs are between those of the ntDNAs and ptDNAs. The ptDNAs can be enriched on the basis of the high abundance of the reads. By contrast, the ntDNAs can be reversely enriched by removing reads with high abundance by using bioinformatic tools, which may have originated from mtDNAs or ptDNAs. However, reads corresponding to mtDNAs cannot be extracted on the basis of their abundance alone, without contaminating ptDNA reads.

**Figure 1:**
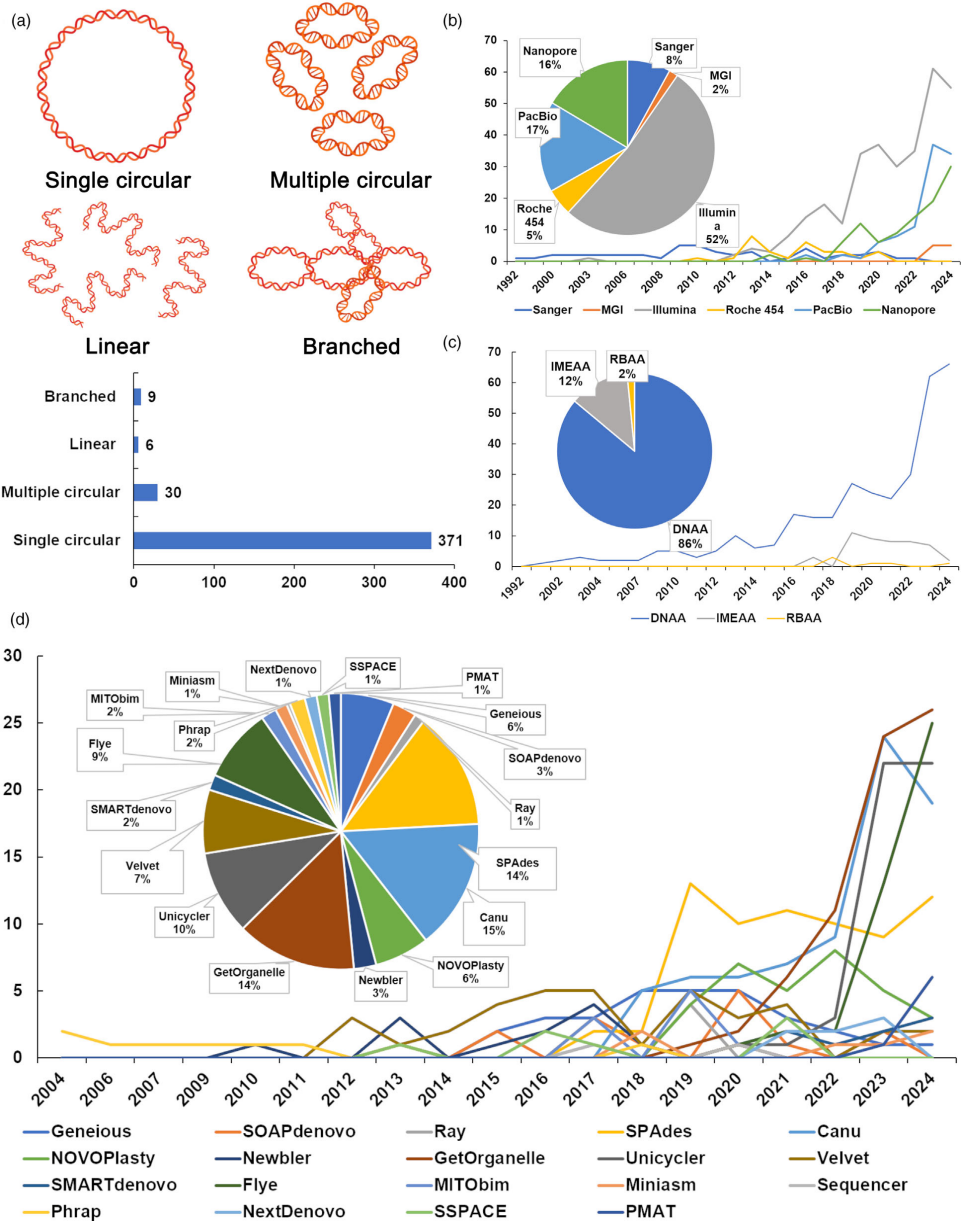
Trends in plant mitochondrial genome structures, sequencing technologies and assembly algorithms. (a) Schematic showing various reported structures of plant mitochondrial genomes. Circular: Single loop of double‐stranded DNA. Multiple circulars: Several independent circular DNA molecules. Linear: Single linear DNA molecules. Branched: Complex DNA topology with branch points. (b) Number of published articles employing different sequencing technologies year by year. The *x*‐axis represents the years, and the *y*‐axis denotes the number of articles published. Sequencing technologies are colour‐coded and explained above the plot. (c) Number of articles using different assembly algorithms by year. The *x*‐axis represents the years, and the *y*‐axis denotes the number of algorithms used. (d) Number of articles using different assemblers' year by year. The *x*‐axis represents the years, and the *y*‐axis represents the number of articles reported to have used the corresponding assembler.

Second, plant mitochondrial genome structures are minimally conserved in terms of size and structure at the interspecific level in the three genomes of plant cells (Gualberto and Newton, 2017; Kozik *et al*., 2019; Wynn and Christensen, 2019). The size of the plant mitochondrial genomes ranged from 66 kb in *Viscum scurruloideum* to 18.99 Mb in *Cathaya argyrophylla* (Huang *et al*., 2024). The configurations of plant mitochondrial genomes include circular, linear and branched mtDNA molecules (Figure 1a). As a result, the plant mitochondrial genome structures cannot be deduced from the plant mitochondrial genomes of closely related species.

Third, plant mitochondrial genome sequences are not conserved at the intergenic regions at the interspecific level, although the gene coding regions are highly conserved. Consequently, the reads corresponding to those of the intergenic regions of mtDNAs cannot be enriched in accordance with their sequence similarity to reference plant mitochondrial genome sequences.

Fourth, the structures of mtDNAs are highly dynamic, which means that the plant mitochondrial genomes from an individual plant may contain multiple configurations, including a major configuration and multiple minor configurations. The phenomena have been described before (Cho *et al*., 2022; Fang *et al*., 2021; Sullivan *et al*., 2020; You *et al*., 2022; Zhang *et al*., 2023) and confirmed in our previous studies, listed in Note S1. Assembling the plant mitochondrial genomes from DNAs extracted from an individual plant requires determining not only the major configuration but also the minor configurations.

Fifth, mtDNAs are known to exchange DNAs with those of nuDNAs and ptDNAs frequently. On the one hand, mtDNA fragments are transferred to nuDNAs, generating the so‐called nuclear mitochondrial DNAs (NUMTs). On the other hand, ptDNAs are frequently transferred to mtDNAs, generating the so‐called mitochondrial plastid DNAs (MTPTs). When assembling plant mitochondrial genomes by using reads from total DNAs, the reads from NUMTs and MTPTs frequently interfere with the process, leading to incorrectly assembled plant mitochondrial genomes.

Lastly, the assembly of plant mitochondrial genomes is complicated by the presence of abundant repetitive sequences. When repetitive sequences exceed the sequencing reads in length, correctly merging reads is a challenge to assembly software tools. The resulting misassembly could lead to misestimation of genome size and the structure of plant mitochondrial genomes.

### Current status of plant mitochondrial genome assembly

Mitochondrial genome‐related literatures were searched using two methods to determine the current status of plant mitochondrial genome research. This work focused on the reported plant mitochondrial genome structures, the sequencing technologies, and the methods and tools used to assemble plant mitochondrial genomes. First, the literature database called Web of Science (https://www.webofscience.com/wos/) was searched using the keywords ‘complete’, ‘mitochondrial’ and ‘genome’ in the title and ‘plant’ as a keyword in all fields on 10 September 2024. The search yielded a total of 757 articles. After these articles were reviewed, 162 articles that reported the sequencing, assembly, annotation and characterization of novel plant mitochondrial genomes were identified.

Second, public sequence databases were searched to find all plant mitochondrial genome sequences, and then the articles describing them were found. All plant mitochondrial genome sequences available in the RefSeq database (10 September 2024) were downloaded. Among them, 688 mitogenomes were from the Viridiplantae clade. By using the Latin names associated with these plant mitochondrial genome sequences, in combination with keywords such as ‘mitochondrial genome’, ‘mitochondrion’ and ‘mtDNA’, the Web of Science database, Google Scholar (https://scholar.google.com/) and PubMed (https://pubmed.ncbi.nlm.nih.gov/) were searched to locate the associated articles. Eventually, a total of 816 articles were found.

After duplicated articles found using the two methods were removed, a total of 741 articles were obtained. Among them, 416 articles described the sequencing, assembly, annotation and characterization of plant mitochondrial genomes. These 416 articles were analysed carefully, and information on the plant mitochondrial genomes' structures, sequencing technologies, assembly algorithms and assembly software tools was extracted. Details are provided in Table S1.

### Plant mitochondrial genome structures

Among these 416 articles, 371, 30 and 15 described the plant mitochondrial genomes as a single circular DNA molecule, multiple circular DNA molecules, and linear and branched DNA molecules, respectively (Figure 1a and Table S1). These studies suggested that approximately 10.8% of the plant mitochondrial genomes are not represented by a single circular DNA molecule. A notable detail is that this number may be an overestimate. The complex structures of some plant mitochondrial genomes may result from incomplete assembly of singular circular DNA molecules.

### High‐throughput DNA sequencing data used for assembling plant mitochondrial genomes

The 416 articles can be divided into three groups on the basis of DNA sequencing technologies. From 1992 to 2010, sequencing was predominantly conducted using Sanger sequencing technology (Wang *et al*., 2024). From 2012 to 2017, the short‐read and Roche 454 technologies were used increasingly for plant mitochondrial genome assembly, with few studies starting to use the Nanopore or PacBio sequencing technologies. From 2018 to 2024, a significant shift was observed, featured by the near‐complete disappearance of the Sanger and Roche 454 sequencing technologies, accompanied by the widespread uses of Nanopore, PacBio and short‐read technologies (Figure 1b). Details are provided in the Table S2 and Note S2.

### Algorithms used for plant mitochondrial genome assembly

Among the 416 articles, 387 articles described the assembly algorithms that were used. These algorithms can be categorized into three major groups: reference‐based assembly algorithm, de novo assembly algorithm and iterative mapping and assembly algorithm. Details of these algorithms are described in the following sections. In total, six studies used RBs, 333 studies used de novo assembly algorithm, and 48 studies used IMEs (Figure 1c and Table S3). Data showed that de novo assembly algorithm has been the predominant algorithm used for plant mitochondrial genome assembly.

### Assemblers used for plant mitochondrial genome assembly

Several assemblers, such as Canu, Flye, SMARTdenovo, Nextdenovo, Velvet and Unicycler, which were initially designed for nuclear genomes and plastomes, were repurposed for assembling plant mitochondrial genomes. Details are provided in Table S4. Several tools were developed to particularly assemble plant mitochondrial genomes. Before 2017, most available assemblers primarily relied on short reads to assemble plant mitochondrial genomes. However, over time, more tools with new algorithms that utilize long reads were developed to improve assembly quality (Figure 1d). For example, four software tools, including GetOrganelle (Jin *et al*., 2020), NOVOPlasty (Dierckxsens *et al*., 2017), Norgal (Al‐Nakeeb *et al*., 2017) and MITObim (Hahn *et al*., 2013), were developed to assemble plant mitochondrial genomes from short‐read data only. By contrast, Plastid and Mitochondrial Assembly Tool (PMAT) (Bi *et al*., 2024) and MitoHiFi (Uliano‐Silva *et al*., 2023) were developed to assemble plant mitochondrial genomes from long‐read data only, such as Pacbio HiFi data. Lastly, GSAT (He *et al*., 2023) and SAGBAC (Fischer *et al*., 2022) were developed to assemble plant mitochondrial genomes by using short‐ and long‐read data simultaneously. Recently, TIPPo (Xian *et al*., 2025) and Oatk (Zhou *et al*., 2024) were developed for assembling plant organelle genomes. TIPPo is a reference‐free organelle genome assembler that uses PacBio HiFi long reads, employing deep learning techniques for read classification and k‐mer counting to enhance the assembly correctness while reducing nuDNA contamination. Oatk software utilized PacBio HiFi data and employed closed syncmers and sparse de Bruijn graph techniques for organelle genome assembly. Oatk can substantially enhance assembly correctness and optimize error correction and sequence annotation.

## Plant mitochondrial genome assembly algorithms

As described above, the genome assembly algorithms suitable for plant mitochondrial genomes can be divided into three groups: reference‐based assembly algorithm, de novo assembly algorithm and iterative mapping and assembly algorithm. Reference‐based assembly algorithm utilized reference genomes to extract reads from the raw sequencing data and then assemble them locally or globally (Liu *et al*., 2018). De novo assembly algorithm assembled the genome without reference sequences. It is most suitable for assembling plant mitochondrial genomes without appropriate reference from closely related species (Liao *et al*., 2019). Iterative mapping and assembly algorithm employed a cyclical process of mapping reads to seed or reference sequences and extending the assembly on the basis of overlapping reads. The process continued until the end could not be extended further (Hahn *et al*., 2013). The following text describes the mechanism of these algorithms and their advantages and disadvantages.

### Reference‐based assembly algorithm

Reference‐based assembly algorithm assembles a genome locally or globally in four steps (Tamazian *et al*., 2016). A schematic representation is shown in Figure S1A. First, a reference genome was selected from a species closely related to the target species. Second, alignment software tools like BWA (Li and Durbin, 2009) or Bowtie (Langmead and Salzberg, 2012) were used to map the sequencing reads to the reference genome. Third, the mapped reads were assembled into contiguous sequence fragments. Lastly, the gaps were filled to obtain the complete genome (de Sa *et al*., 2016). Six of the plant mitochondrial genome articles employed this algorithm. Details are provided in Table S1.

Reference‐based assembly algorithm has two main advantages. First, extracting reads mapped to the reference genome for further assembly could reduce the input data's size and remarkably lower the requirement of computational resources (Liu *et al*., 2018). Second, reference‐based assembly algorithm allows the identification of nucleotide variations, including single nucleotide polymorphisms, insertions and deletions (Chan *et al*., 2016), and it is particularly suited for population genomic studies when the reference genome of a species is available.

Reference‐based assembly algorithm has two potential problems. First, the species with sequenced plant mitochondrial genomes or the availability of potential plant mitochondrial genome reference genomes is relatively minimal (Wang *et al*., 2024). As a result, the plant mitochondrial genomes that can use reference‐based assembly algorithm to assemble are relatively minimal. Second, plant mitochondrial genomes have considerable structural variations, even among individuals from the same species (Gualberto and Newton, 2017). Thus, reference genomes may not be useful for identifying mtDNA reads from the sequencing data.

### De novo assembly algorithm

#### Overview

De novo assembly algorithm should be used when no appropriate reference genome is available. This algorithm has two types: overlap‐layout‐consensus (OLC) and de Bruijn graph. As shown in Figure S1B, the OLC algorithm assembles a genome on the basis of the overlay of sequence reads (Baker, 2012). In the assembly process, reads having overlapping sequences are first connected to form longer sequence fragments, called contigs. Then, larger sequence frameworks, such as scaffolds or chromosomes, are established on the basis of additional information such as pair‐end information (Sohn and Nam, 2018). Lastly, additional methods, such as PCR amplification with primers spanning the gaps, are used to fill the sequence gaps.

For high‐throughput data generated by short‐read technologies, determining the overlay can be computationally expensive by using the OLC algorithm. The de Bruijn graph algorithm has been widely used to overcome this drawback (Rizzi *et al*., 2019). As shown in Figure S1C, this algorithm constructs a graph from short DNA reads. Reads are broken into k‐mers of a fixed length. Nodes in the graph represent unique k‐mers, and edges connect k‐mers with overlapping parts. Genome assembly aims to determine an optimal Eulerian path through the graph, representing the assembled genome. The de novo assembly algorithm can be used to assemble short reads generated by the Illumina platform and long reads generated with PacBio CLR, PacBio HiFi and Oxford Nanopore Technologies (ONTs). The specific characteristics of these sequencing technologies are beyond the scope of this article, and interested readers can refer to the reference article (Scarano *et al*., 2024) for more information. The differences in the performance of these sequencing platforms in mitochondrial genome assembly are discussed in section De novo assembly algorithm using short‐read data only.

##### De novo assembly algorithm using short‐read data only

Among the 416 articles, 90 studies used de novo assembly algorithms to assemble short‐read data. These 90 articles are listed in Table S1. Short‐read technologies are cost‐effective and have higher correctness than long‐read technologies. However, short‐read data are usually shorter than the repetitive sequences found in plant mitochondrial genomes. As a result, short‐read data are unable to resolve the repetitive regions. Experimentally, using a mate‐pair library of mtDNAs could solve the problems because mate‐pair reads can show relationships of reads up to 50 kb apart (Nagarajan and Pop, 2013). However, constructing mate‐pair libraries requires high‐quality DNAs, which are difficult to obtain for mtDNAs. In addition, given that mtDNAs are usually intermingled with nuDNAs and ptDNAs, constructing mate‐pair libraries requiring sufficient template DNAs from mtDNAs becomes even more difficult. Taken together, the main drawback of short‐read data is that they may not be able to resolve the repetitive regions.

##### De novo assembly algorithm using Long‐read data only

As long‐read sequencing technologies, such as PacBio and ONT, develop rapidly, they gradually become the mainstream technologies to sequence total DNAs for plant mitochondrial genome assembly. Long‐read sequencing libraries are relatively easy to construct compared with mate‐pair libraries, and the resulting long reads are usually longer than the repetitive regions. Theoretically, longer reads provide greater assistance in genome assembly, and the ideal scenario is to achieve single‐molecule sequencing of the mitochondria. For instance, nanopore sequencing can currently generate reads as long as 4 Mb, which can cover the mitochondrial genomes of most species. However, in practical applications, current sequencing platforms still fall short of this capability in mitochondrial genome sequencing.

In addition to read length, error rate is an important standard for mitochondrial genome research of long reads. Currently, long reads sequencing platforms can be categorized into PacBio HiFi and others on the basis of their error rates. The PacBio HiFi sequencing technology was developed by PacBio. It utilizes circular consensus sequencing to produce reads that are ~10–25 kb, with low error rates of ~0.1–0.5%, similar to those of the Illumina short‐read technologies. By contrast, ONT can generate reads as long as 4 Mb, making it ideal for processing complex genomes with extensive repetitive sequences. ONT's advantage in long reads allows it to span longer repetitive regions, whereas HiFi's high‐correctness reads reduce the need for subsequent error correction. Each technology has its strengths, and the choice should be based on specific research needs and genomic characteristics.

Among the 416 articles, 40 utilized Nanopore or PacBio data only. Details are provided in Table S1. The main drawback of the long‐read sequencing technologies at present is that their reads usually have an error rate higher than those of short‐read technologies, frequently leading to erroneous plant mitochondrial genome assembly.

##### De novo assembly algorithm using short‐ and long‐read data

Short‐ data and long‐read data are complementary to each other in terms of read length and error rate. Several articles assembled plant mitochondrial genomes by utilizing short‐ and long‐read data. These articles are listed in Table S1. So far, three different de novo assembly methods have been developed. The first method is called de novo‐long‐read‐sequencing‐assembly‐short‐read‐Polish, or ‘de novo‐short/long method 1’ in short. For this method, a plant mitochondrial genome was first de novo assembled using long‐read data. Then, the assembled contig sequences were subjected to error correction by mapping the short‐read data to the assembled contig sequences. The drawback of this method is that it may erroneously correct a region with reads from an unexpected region. During error correction of mitochondrial plastid transferred regions (MTPT) in plant mitochondrial genomes, a risk of mistakenly including chloroplast genome reads exists, potentially leading to incorrect assembly. In total, 90 articles employed this method. Two other methods, the second and the third methods, did not involve polishing.

**Figure 2:**
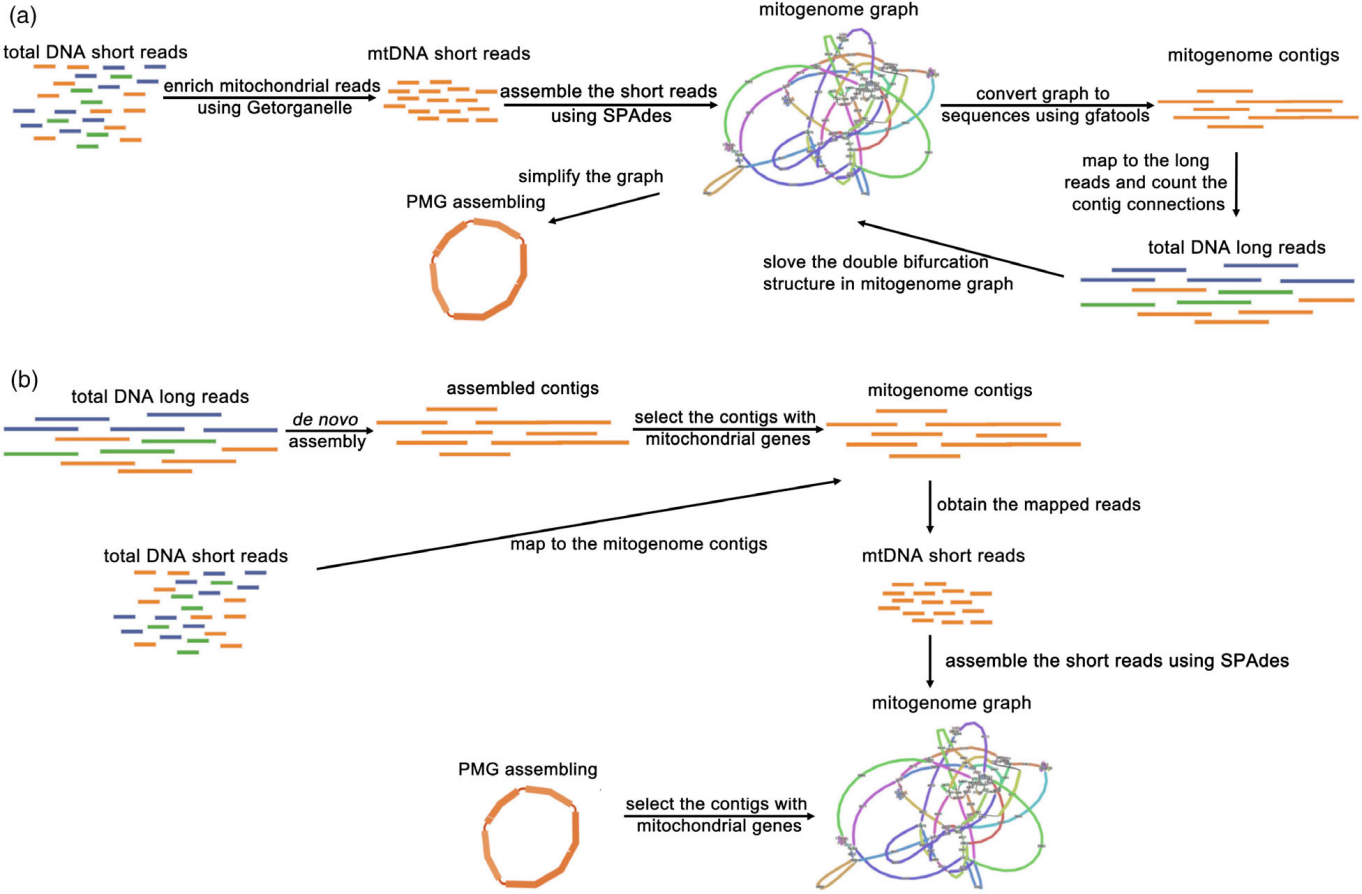
Two workflows for assembling plant mitochondrial genomes without polishing by using short‐ and long‐read data. (a) In the first workflow, short‐read data from mtDNAs are enriched. These reads are subsequently assembled to obtain a preliminary assembly graph. The sequences corresponding to the graph are extracted and aligned against the long‐read sequencing reads. Subsequently, the corresponding double bifurcation structures in the graph are resolved, resulting in the final plant mitochondrial genome assembly. (b) In the second workflow, long‐read data of total DNA are assembled de novo. Mitogenome contigs, identified by the presence of mitochondrial genes, are extracted from the assembly results. Afterwards, short‐read reads are aligned to these mitochondrial contigs. The aligned reads are then integrated into a genome graph. This graph undergoes the same process to resolve the double bifurcation structure as described in (a).

The second method is called de novo‐short‐assembly/long‐read‐selection, or ‘de novo‐short/long method 2’ in short. For this method, the short‐read data were assembled into a graph, and then the long‐read data were mapped to the graph. The paths supported by abundant long‐read sequencing reads were kept, generating the final assembly (Figure 2a).

The third method, called ‘de novo ‐short/long method 3’, is a variation of method 2. For this method, the long‐read data were first assembled into contigs. Plant mitochondrial genome sequences from public databases were then compared with these contigs, and those contigs similar to the plant mitochondrial genome sequences were selected as plant mitochondrial genome contigs. The short‐read data were mapped to these plant mitochondrial genome contigs, and those mapped short‐read data were assembled into an assembly graph. Lastly, the long‐read data were mapped to the graph. Those paths supported with most long‐read data were selected, resulting in a simplified assembly (Figure 2b). Among the 333 articles with de novo, 78 utilized either method 2 or method 3. The exact method could not be determined because some articles did not provide sufficient details of the assembly process (Table S1).

#### Advantages of de novo assembly algorithm

De novo assembly algorithm does not rely on any reference plant mitochondrial genomes, thus avoiding potential biases caused by using the reference plant mitochondrial genomes. In addition, this algorithm allows the uncovering of unique genomic features of the new plant mitochondrial genome, including new genes or variations that may be absent in the reference plant mitochondrial genomes (Baker, 2012). De novo is particularly effective in detecting large‐scale genomic structural variations, such as insertions, deletions and inversions, which may be challenging to identify using reference‐based assembly algorithm (Chu *et al*., 2013).

#### Disadvantages of de novo assembly algorithm

De novo assembly algorithm has several disadvantages. First, it demands considerably more computational resources than reference‐based assembly algorithm, particularly when dealing with large genomes (Sohn and Nam, 2018). Second, in the absence of a reference genome, the assembly process is highly susceptible to influences from factors such as read quality, read length and the abundance of repetitive sequences. These factors collectively increase the assembly error rate (Schmid *et al*., 2018). Achieving high‐quality de novo plant mitochondrial genome assemblies requires substantial sequencing data to ensure sufficient coverage depth. The success of de novo assembly algorithm is largely contingent upon the performance of the assembly tools, which exhibit variability in their adaptability to plant mitochondrial genomes from different species (Desai *et al*., 2013). In a whole‐genome sequencing experiment, reads from the plant mitochondrial genomes constituted a small proportion of the total data only. Plant mitochondrial genome assembled with de novo assembly algorithm could inadvertently include nuDNA and ptDNA fragments, leading to erroneous plant mitochondrial genome assembly (Figure 2b).

### Iterative mapping and extension

Iterative mapping and assembly algorithm progressively maps reads to a seed sequence, extends the end of the seed and then repeats the above process through multiple iterations (Figure S1D) (Tsai *et al*., 2010). Each iteration updates and optimizes the assembly on the basis of the results of the previous mapping and extension until the desired coverage and correctness are achieved. Among 416 articles, 49 used iterative mapping and assembly algorithm, as listed in Table S1.

Iterative mapping and assembly algorithm has two advantages over de novo assembly algorithm. First, it uses less computational resources. Iterative mapping and assembly algorithm uses the reads mapped to the end of the seed sequence to assemble and extend the seed sequence. Usually, only a small fraction of all reads are mapped to the end of the seed sequences, and assembling these mapped reads requires significantly less computational resources (Choudhury *et al*., 2018). Second, it allows error correction at each step. In each iteration, new reads mapped to the ends of the seed sequences can be utilized to rectify potential errors left from previous iterations. This step can improve the overall correctness of the assembly (Otto *et al*., 2010).

Iterative mapping and assembly algorithm has three disadvantages. First, it requires a high‐quality seed sequence to start. Using different seeds may lead to different assembly results (Westbury and Lorenzen, 2022). Second, it stops extending the end when encountering a sequencing gap, leading to partial plant mitochondrial genome assembly. Lastly, in cases of multiple options to extend the end of the seed, the iterative mapping and assembly algorithm tends to select one of them only on the basis of selection criteria and ignores other paths that may represent the plant mitochondrial genome's actual configurations (Wang *et al*., 2019).

## Plant mitochondrial genome assemblers

The usage frequencies of the plant mitochondrial genome assemblers in the 416 articles were calculated (Table S1). The year‐by‐year usage of the assemblers like Velvet, Sequencer and Flye (Kolmogorov *et al*., 2019) varies significantly. Beginning in 2021, Flye, Canu and GetOrganelle have been increasingly used to assemble plant mitochondrial genomes. Among them, GetOrganelle and Canu are the two most popular assemblers and have been used in 32 and 33 studies, respectively (Table S3). The most frequently used assemblers are described below. Some other less used assemblers, such as MITObim, Norgal, MitoHiFi, Organelle‐PBA, SAGBAC and GSAT, are described in Note S3. These assemblers are grouped in accordance with the type of sequencing data they use.

### Plant mitochondrial genome assemblers that use short‐read data only

#### GetOrganelle

GetOrganelle utilizes the de novo assembly algorithm (Table 1) (Jin *et al*., 2020). It is widely used for assembling various organellar genomes, including plant plastid genomes (Mo *et al*., 2022; Wang *et al*., 2022; Yichao *et al*., 2021; Zhou *et al*., 2021), animal mitochondrial genomes (Baeza *et al*., 2023; Cronin *et al*., 2022; Wang *et al*., 2024; Xing *et al*., 2023) and fungal mitochondrial genomes (Blanco Casallas, 2024; Li *et al*., 2023; Shao *et al*., 2020; Zhang *et al*., 2022). It has also been used to construct the initial assembly graph of plant mitochondrial genomes (Braich *et al*., 2020; De Abreu *et al*., 2023; Li *et al*., 2021; Yu *et al*., 2023). GetOrganelle utilizes SPAdes as the assembly engine and has added several optimization steps. For example, it can enrich organellar reads on the basis of the specified reference sequences. Previous studies showed that GetOrganelle performed the best in assembling plastid genomes (Freudenthal *et al*., 2020). However, GetOrganelle cannot easily assemble plant mitochondrial genomes, primarily due to the presence of numerous repetitive sequences in the plant mitochondrial genomes and the contaminating reads from nuDNAs and ptDNAs. Although some chloroplast fragments can be removed on the basis of their high coverage depth, those mitochondrial fragments containing regions similar to the plastid genomes may be mistakenly removed at the same time. Literature analysis showed that it has been used 69 times in the past 5 years, making it the most frequently used software tool for plant mitochondrial genome assembly (Table 2).

**Table 1:** List of commonly used plant mitochondrial genome assemblers

Software name	Type of data accepted	Algorithm	Implementing language	GitHub repository
GetOrganelle	Short reads only	de novo	Python	https://github.com/Kinggerm/GetOrganelle
NOVOPlasty	Short‐read only	IME	Perl	https://github.com/ndierckx/NOVOPlasty
Norgal	Short‐read only	RB	Java	https://bitbucket.org/kosaidtu/norgal/src/master/
MITObim	Short‐read only	IME	Perl	https://github.com/chrishah/MITObim
OrganellePBA	Pacbio only	RB	Perl	https://github.com/ndierckx/NOVOPlasty
MitoHiFi	Pacbio HiFi only	de novo	Python	https://github.com/marcelauliano/MitoHiFi
PMAT	Long‐read sequencing only	de novo	Python	https://github.com/bichangwei/PMAT
GSAT	Short‐read + long‐read sequencing	de novo	Perl	https://github.com/hwc2021/GSAT
SAGBAC	Short‐read + long‐read sequencing	de novo	R	https://github.com/AxelMacFoly/SAGBAC

IME, iterative mapping and extension; RB, reference‐based assembly algorithm.

**Table 2:** Ease setup of installation table

Number of PMGATs	Name of PMGATs	Easy installation by Conda	Test data availability	Manual availability	Type of data accepted
1	GetOrganelle	Yes	Yes	Yes	Short reads only
2	Velvet	Yes	No	Yes	Short reads only
3	NOVOPlasty	Yes	No	Yes	Short reads only
4	SOAPdenovo2	Yes	No	Yes	Short reads only
5	Canu	Yes	No	Yes	Long reads only
6	Flye	Yes	No	Yes	Long reads only
7	SMARTdenovo	Yes	No	Yes	Long reads only
8	PMAT	No	Yes	Yes	Long reads only
9	NextDenovo	Yes	No	Yes	Long reads only
10	SPAdes	Yes	No	Yes	Short reads only, long reads only, and short and long reads
11	Unicycler	Yes	No	Yes	Short reads only, long reads only, and short and long reads
12	TIPPo	Yes	No	Yes	High‐accuracy long reads
13	Oatk	Yes	No	Yes	High‐accuracy long reads

The test data and manual were sorted in the following links: (1) https://github.com/Kinggerm/GetOrganelle, (2) https://github.com/dzerbino/velvet, (3) https://github.com/Edith1715/NOVOplasty, (4) https://github.com/aquaskyline/SOAPdenovo2, (5) https://github.com/marbl/canu, (6) https://github.com/mikolmogorov/Flye, (7) https://github.com/ruanjue/smartdenovo, (8) https://github.com/bichangwei/PMAT, (9) https://github.com/Nextomics/NextDenovo, (10) https://github.com/ablab/spades, (11) https://github.com/rrwick/Unicycler, (12) https://github.com/Wenfei‐Xian/TIPP, (13) https://github.com/c‐zhou/oatk.

#### NOVOPlasty

NOVOPlasty utilizes the iterative mapping and assembly algorithm (Table 1) (Dierckxsens *et al*., 2017). This tool initially stores sequence data in a hash table, allowing rapid access to read sequences. The assembly process starts with a seed sequence and proceeds with bidirectional extension iteratively. NOVOPlasty supports a wide range of input seed sequences. However, the choice of seed sequence could affect the assembly outcomes. NOVOWrap is the successor of NOVOPlasty, and it supports the use of multi‐seed sequences (Wu *et al*., 2021). It has been used for 40 times in the past 5 years (Table 2).

#### Velvet

Velvet utilizes the de Bruijn graph algorithm, which belongs to short‐read de novo assembly (Zerbino and Birney, 2008). Velvet breaks each read sequence into k‐length subsequences, also known as k‐mers, which are then used to construct a de Bruijn graph representing the relationships among all the read sequences. Following the construction of the graph, a series of graph simplification processes is applied to reduce complexity and correct for sequencing errors. Ultimately, continuous sequence paths are extracted from the simplified graph, resulting in the generation of contig sequences. It was used 11 times in the past 5 years (Table 2).

#### SOAPdenovo

SOAPdenovo utilizes the de Bruijn graph algorithm (Luo *et al*., 2012). It uses a multiple k‐mer strategy to handle the repetitive regions. It was used eight times in the past 5 years (Table 2 and Table S1).

#### Disadvantages of plant mitochondrial genome assemblers using short‐read data only

Despite the progress achieved by tools like GetOrganelle, NOVOPlasty, Velvet, SOAPdenovo, Norgal and MITObim, assembling plant mitochondrial genomes by using short‐read data alone has several disadvantages. First, the length of short‐read data is too short to span mitochondrial repetitive regions, and thus, the bubbles in the assembly graph cannot be resolved. Short reads from regions showing a high level of similarity among mtDNAs and nuDNAs and ptDNAs could not be separated from each other, consequently leading to difficulty in assembling these regions correctly.

### Plant mitochondrial genome assemblers that use Long‐read data only

#### Canu

Canu utilizes the OLC algorithm to assemble long‐read data. First, it corrects the long‐read data's errors. Then, it employs the tf‐idf weighted MinHash algorithm to detect overlapping regions, facilitating the construction of the target genome. Canu was not originally tailored for plant mitochondrial genome assembly. Among the 416 studies, Canu was utilized 32 times in the past 5 years.

#### Flye

Flye utilizes the de Bruijn graph algorithm to assemble long‐read data (Kolmogorov *et al*., 2019). Flye constructs accurate repeat graphs from error‐prone data segments called disjointigs and builds the assembly graph. Flye was not originally designed for plant mitochondrial genome assembly. Among the 416 plant mitochondrial genome studies, Flye was used 25 times in the past 5 years.

#### SMARTdenovo

SMARTdenovo utilizes the de novo assembly algorithm to assemble long‐read data (Liu *et al*., 2021). It develops a Best‐Overlap‐Graph and PBDAG‐Con algorithm, which uses homopolymer‐compressed k‐mers for seed indexing, read layout optimization and consensus sequence generation. This software was cited nine times in the past 5 years (Table 2 and Table S1).

#### PMAT

PMAT uses Canu as the assembly engine. After the initial assembly is completed, PMAT selects contigs with gene‐coding sequences from the assembly results to construct an assembly graph. The final results could be visualized using Bandage software to manually determine the final assembly (Bi *et al*., 2024).

#### NextDenovo

NextDenovo utilizes a modified OLC algorithm to assemble long‐read data (Hu *et al*., 2024). Initially, NextDenovo performs multiple‐sequence alignments. Then, error correction is performed on the basis of the alignment. It then identifies overlapping regions among reads to form an overlap graph, which represents the read relationships. Finally, NextDenovo parses the graph into a continuous genomic sequence. NextDenovo was cited seven times in the past 5 years (Table 2 and Table S1).

#### Challenges in assembling plant mitochondrial genomes by using Long‐read data alone

While long reads are advantageous over short reads in terms of length, they have the disadvantage of higher error rates (Amarasinghe *et al*., 2020). Consequently, correcting errors in raw long reads is crucial for the downstream assembly process. Many error correction algorithms have been developed (Mitchell *et al*., 2020). The effects of their processing are difficult to evaluate. Improvements in sequencing technologies or error correction algorithms are urgently needed.

### Plant mitochondrial genome assemblers that use short‐ and Long‐read data simultaneously

Four assemblers, including SPAdes, GSAT, SAGBAC and Unicycler, were used to assemble plant mitochondrial genomes with short‐read and long‐read data. Two of the most used tools are described below.

#### SPAdes

SPAdes utilizes the de novo assembly algorithm to assemble short‐ and long‐read data (*de novo*‐short/long method 2) (Prjibelski *et al*., 2020). It first assembles short‐read data and constructs a de Bruijn graph. Then, it uses long‐read data to bridge areas that short‐read data cannot cover, ultimately obtaining a reference genome. It closes the gaps in the assembly graph by using the consensus of long‐read data that span the gaps. SPAdes was cited 52 times in the past 5 years (Table 2 and Table S1).

#### Unicycler

Unicycler utilizes the de novo assembly algorithm to assemble short‐ and long‐read data (de novo‐short/long method 2) (Wick *et al*., 2017). Unicycler assembles a genome in the following steps. First, Unicycler uses SPAdes to perform de novo assembly of short‐read data. Unicycler adjusts the k‐mer sizes to optimize the assembly graph and minimize unconnected end regions (Figure S2) (Bankevich *et al*., 2012). Second, on the basis of coverage depth and graph structural information, Unicycler uses a greedy algorithm to assign copy numbers to different contigs. Unicycler compares the connected contigs in the short‐read data assembly graph with long‐read data to simplify the assembly graph, a process called bridging. The adjacent bridging regions are merged to form continuous structures (Figure S3) (Walker *et al*., 2014). Unicycler was cited 50 times in the past 5 years (Table 2 and Table S1).

## Evaluation of the performance of plant mitochondrial genome assemblers

### Selection criteria for plant mitochondrial genome assembly tools

All software tools were ranked on the basis of their citation numbers between 2019 and 2024, and those ranked at the top 10 positions were selected for further evaluation. Eleven tools were selected, including GetOrganelle (Jin *et al*., 2020), Velvet (Zerbino and Birney, 2008), NOVOPlasty (Dierckxsens *et al*., 2017), SOAPdenovo2 (Luo *et al*., 2012), Canu (Koren *et al*., 2017), Flye (Kolmogorov *et al*., 2019), SMARTdenovo (Liu *et al*., 2021), PMAT (Bi *et al*., 2024), NextDenovo (Hu *et al*., 2024), SPAdes (Bankevich *et al*., 2012) and Unicycler (Wick *et al*., 2017). The last two tools shared the 10th position (Table 2). Two most recently published tools, TIPPo and Oatk, were included considering that they may represent the most advanced tools but do not have sufficient citation numbers because they were published recently. In summary, a total of 13 tools were used for evaluation and comparison.

**Figure 3:**
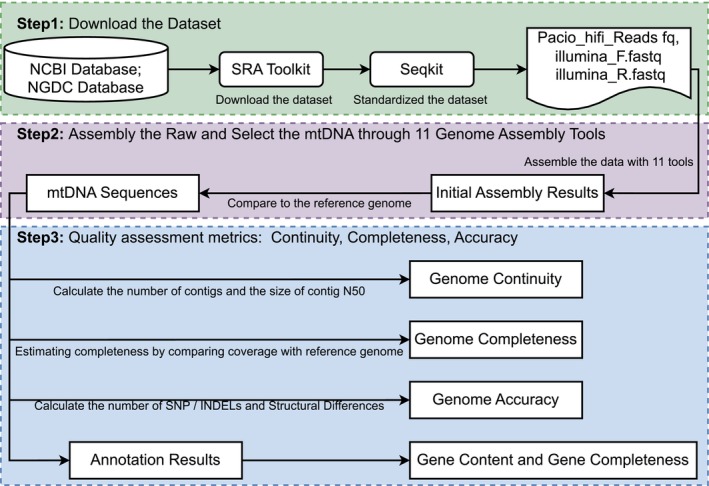
Workflow for evaluating plant mitochondrial genome assemblers. Step 1 involves downloading and standardizing the dataset from NCBI and NGDC databases by using SRA Toolkit and Seqkit to prepare the data for assembly. In step 2, the mtDNA sequences are extracted and assembled using 11 different genome assembly tools, followed by a comparison to the reference genome to produce initial assembly results. Step 3 focuses on quality assessment metrics, including genome contiguity, completeness and correctness. Contiguity is assessed by calculating the number of contigs and the size of contig N50. Completeness is estimated by comparing the coverage of the assembled genome with the reference genome. Correctness is evaluated by calculating the number of SNPs, INDELs and structural differences, leading to annotation results that include gene content and gene completeness.

Each tool contains a wide range of adjustable parameters, and the optimal settings may vary depending on the characteristics of the samples. Defining a single best configuration is not feasible. All tools were tested using their default parameters to ensure a fair cross‐tool benchmarking. The workflow to evaluate the performance of these tools is shown in Figure 3. Some tools had some particular requirements to run. For example, Canu required the specification of a genome size of a reference genome for assembly. PMAT integrated Canu in its pipeline, so it had the same requirement. NOVOPlasty required a seed sequence to be specified in its configuration file for organellar genome assembly. The configuration file is provided in Appendix S1. NextDenovo was originally configured for nuclear genome assembly, and it generated errors when assembling organellar genomes. Consequently, its configuration was modified to enable accurate organellar genome assembly. The configuration file is provided in Appendix S2. Regarding the type of supported sequencing data, GetOrganelle, Velvet, NOVOPlasty and SOAPdenovo2 were designed to assemble Illumina data. By contrast, Canu, Flye, SMARTdenovo, PMAT and NextDenovo were optimized to assemble PacBio data. SPAdes and Unicycler supported hybrid assembly of Illumina and PacBio data.

The tools setup for all software can be found in Notes S4 and S5. Several tools do not differentiate contigs from the nuclear, mitochondrial and chloroplast genomes. A method to extract mitochondrial contigs from the contigs of all three genomes was developed to fairly evaluate the accuracy of mitochondrial genome assembly by these tools. These mitochondrial contigs were retained for further analyses. Details of the method can be found in Note S5.

### Dataset used

The sequencing datasets of two model species, *A. thaliana* and *O. sativa*, were selected for evaluation. The assembly results from only the short and long reads obtained from the same biological sample were compared to minimize inter‐individual variations of the plant mitochondrial genome. In particular, the dataset for *A. thaliana* was obtained from two studies (Kang *et al*., 2023; Wang *et al*., 2021), containing data from four different individual plant samples (Table 3), whereas the data for *O. sativa* came from one study only (Zhang *et al*., 2022), containing data from four different individual plant samples. The Sequence Read Archive accession numbers for these datasets are provided in Table 3. The correspondence among sample names, data types and accession numbers used for each assembly run is provided in Table 3.

**Table 3:** List of correspondence among sample names, data types and associated run accession numbers

Run no.	Software	Species	Sample no.	Dataset no.	Sub‐dataset no.	SRA accession no.
*A. thaliana*	Canu	Run‐001	Sample 1	Dataset‐1	sub‐dataset‐1‐ Pacbio	CRR302668
		Run‐002	Sample 2	Dataset‐2	sub‐dataset‐2‐ Pacbio	CRR339253
		Run‐003	Sample 3	Dataset‐3	sub‐dataset‐3‐ Pacbio	CRR591658
		Run‐004	Sample 4	Dataset‐4	sub‐dataset‐4‐ Pacbio	CRR591657
	Flye	Run‐005	Sample 1	Dataset‐1	sub‐dataset‐1‐ Pacbio	CRR302668
		Run‐006	Sample 2	Dataset‐2	sub‐dataset‐2‐ Pacbio	CRR339253
		Run‐007	Sample 3	Dataset‐3	sub‐dataset‐3‐ Pacbio	CRR591658
		Run‐008	Sample 4	Dataset‐4	sub‐dataset‐4‐ Pacbio	CRR591657
	GetOrganelle	Run‐009	Sample 1	Dataset‐1	sub‐dataset‐1‐ Illumina	CRR302670
		Run‐010	Sample 2	Dataset‐2	sub‐dataset‐2‐ Illumina	CRR338532
		Run‐011	Sample 3	Dataset‐3	sub‐dataset‐3‐ Illumina	CRR624284
		Run‐012	Sample 4	Dataset‐4	sub‐dataset‐4‐ illumina	CRR624285
	NextDenovo	Run‐013	Sample 1	Dataset‐1	sub‐dataset‐1‐ Pacbio	CRR302668
		Run‐014	Sample 2	Dataset‐2	sub‐dataset‐2‐ Pacbio	CRR339253
		Run‐015	Sample 3	Dataset‐3	sub‐dataset‐3‐ Pacbio	CRR591658
		Run‐016	Sample 4	Dataset‐4	sub‐dataset‐4‐ Pacbio	CRR591657
	PMAT	Run‐017	Sample 1	Dataset‐1	sub‐dataset‐1‐ Pacbio	CRR302668
		Run‐018	Sample 2	Dataset‐2	sub‐dataset‐2‐ Pacbio	CRR339253
		Run‐019	Sample 3	Dataset‐3	sub‐dataset‐3‐ Pacbio	CRR591658
		Run‐020	Sample 4	Dataset‐4	sub‐ dataset‐4‐ Pacbio	CRR591657
	SMARTdenovo	Run‐021	Sample 1	Dataset‐1	sub‐dataset‐1‐ Pacbio	CRR302668
		Run‐022	Sample 2	Dataset‐2	sub‐dataset‐2‐ Pacbio	CRR339253
		Run‐023	Sample 3	Dataset‐3	sub‐dataset‐3‐ Pacbio	CRR591658
		Run‐024	Sample 4	Dataset‐4	sub‐dataset‐4‐ Pacbio	CRR591657
	SOAPdenovo2	Run‐025	Sample 1	Dataset‐1	sub‐dataset‐1‐ Illumina	CRR302670
		Run‐026	Sample 2	Dataset‐2	sub‐dataset‐2‐ Illumina	CRR338532
		Run‐027	Sample 3	Dataset‐3	sub‐dataset‐3‐ Illumina	CRR624284
		Run‐028	Sample 4	Dataset‐4	sub‐dataset‐4‐ Illumina	CRR624285
	Velvet	Run‐029	Sample 1	Dataset‐1	sub‐dataset‐1‐ Illumina	CRR302670
		Run‐030	Sample 2	Dataset‐2	sub‐dataset‐2‐ Illumina	CRR338532
		Run‐031	Sample 3	Dataset‐3	sub‐dataset‐3‐ Illumina	CRR624284
		Run‐032	Sample 4	Dataset‐4	sub‐dataset‐4‐ Illumina	CRR624285
	TIPPo	Run‐033	Sample 1	Dataset‐1	sub‐dataset‐1‐ Pacbio	CRR302668
		Run‐034	Sample 2	Dataset‐2	sub‐dataset‐2‐ Pacbio	CRR339253
		Run‐035	Sample 3	Dataset‐3	sub‐dataset‐3‐ Pacbio	CRR591658
		Run‐036	Sample 4	Dataset‐4	sub‐dataset‐4‐ Pacbio	CRR591657
	Oatk	Run‐037	Sample 1	Dataset‐1	sub‐dataset‐1‐ Pacbio	CRR302668
		Run‐038	Sample 2	Dataset‐2	sub‐dataset‐2‐ Pacbio	CRR339253
		Run‐039	Sample 3	Dataset‐3	sub‐dataset‐3‐ Pacbio	CRR591658
		Run‐040	Sample 4	Dataset‐4	sub‐dataset‐4‐ Pacbio	CRR591657
	NOVOPlasty	Run‐041	Sample 1	Dataset‐1	sub‐dataset‐1‐ Illumina	CRR302670
		Run‐042	Sample 2	Dataset‐2	sub‐dataset‐2‐ Illumina	CRR338532
		Run‐043	Sample 3	Dataset‐3	sub‐dataset‐3‐ Illumina	CRR624284
		Run‐044	Sample 4	Dataset‐4	sub‐dataset‐4‐ Illumina	CRR624285
	SPAdes	Run‐045	Sample 1	Dataset‐1	sub‐dataset‐1‐ Illumina sub‐dataset‐1‐ Pacbio	CRR302670 and CRR302668
		Run‐046	Sample 2	Dataset‐2	sub‐dataset‐2‐ Illumina sub‐dataset‐2‐ Pacbio	CRR338532 and CRR339253
		Run‐047	Sample 3	Dataset‐3	sub‐dataset‐3‐ Illumina sub‐dataset‐3‐ Pacbio	CRR624284 and CRR591658
		Run‐048	Sample 4	Dataset‐4	sub‐dataset‐4‐ Illumina sub‐dataset‐4‐ Pacbio	CRR624285 and CRR591657
	Unicycler	Run‐049	Sample 1	Dataset‐1	sub‐dataset‐1‐ Illumina sub‐dataset‐1‐ Pacbio	CRR302670 and CRR302668
		Run‐050	Sample 2	Dataset‐2	sub‐dataset‐2‐ Illumina sub‐dataset‐2‐ Pacbio	CRR338532 and CRR339253
		Run‐051	Sample 3	Dataset‐3	sub‐dataset‐3‐ Illumina sub‐dataset‐3‐ Pacbio	CRR624284 and CRR591658
		Run‐052	Sample 4	Dataset‐4	sub‐dataset‐4‐ Illumina sub‐dataset‐4‐ Pacbio	CRR624285 and CRR591657
*O. sativa*	Canu	Run‐053	Sample 1	Dataset‐1	sub‐dataset‐1‐ Pacbio	CRR453538
		Run‐054	Sample 2	Dataset‐2	sub‐dataset‐2‐ Pacbio	CRR453541
		Run‐055	Sample 3	Dataset‐3	sub‐dataset‐3‐ Pacbio	CRR453545
		Run‐056	Sample 4	Dataset‐4	sub‐dataset‐4‐ Pacbio	CRR453549
	Flye	Run‐057	Sample 1	Dataset‐1	sub‐dataset‐1‐ Pacbio	CRR453538
		Run‐058	Sample 2	Dataset‐2	sub‐dataset‐2‐ Pacbio	CRR453541
		Run‐059	Sample 3	Dataset‐3	sub‐dataset‐3‐ Pacbio	CRR453545
		Run‐060	Sample 4	Dataset‐4	sub‐dataset‐4‐ Pacbio	CRR453549
	GetOrganelle	Run‐061	Sample 1	Dataset‐1	sub‐dataset‐1‐ Illumina	CRR453540
		Run‐062	Sample 2	Dataset‐2	sub‐dataset‐2‐ Illumina	CRR453544
		Run‐063	Sample 3	Dataset‐3	sub‐dataset‐3‐ Illumina	CRR453548
		Run‐064	Sample 4	Dataset‐4	sub‐dataset‐4‐ Illumina	CRR453552
	NextDenovo	Run‐065	Sample 1	Dataset‐1	sub‐dataset‐1‐ Pacbio	CRR453538
		Run‐066	Sample 2	Dataset‐2	sub‐dataset‐2‐ Pacbio	CRR453541
		Run‐067	Sample 3	Dataset‐3	sub‐dataset‐3‐ Pacbio	CRR453545
		Run‐068	Sample 4	Dataset‐4	sub‐dataset‐4‐ Pacbio	CRR453549
	PMAT	Run‐069	Sample 1	Dataset‐1	sub‐dataset‐1‐ Pacbio	CRR453538
		Run‐070	Sample 2	Dataset‐2	sub‐dataset‐2‐ Pacbio	CRR453541
		Run‐071	Sample 3	Dataset‐3	sub‐dataset‐3‐ Pacbio	CRR453545
		Run‐072	Sample 4	Dataset‐4	sub‐dataset‐4‐ Pacbio	CRR453549
	SMARTdenovo	Run‐073	Sample 1	Dataset‐1	sub‐dataset‐1‐ Pacbio	CRR453538
		Run‐074	Sample 2	Dataset‐2	sub‐dataset‐2‐ Pacbio	CRR453541
		Run‐075	Sample 3	Dataset‐3	sub‐dataset‐3‐ Pacbio	CRR453545
		Run‐076	Sample 4	Dataset‐4	sub‐dataset‐4‐ Pacbio	CRR453549
	SOAPdenovo2	Run‐077	Sample 1	Dataset‐1	sub‐dataset‐1‐ Illumina	CRR453540
		Run‐078	Sample 2	Dataset‐2	sub‐dataset‐2‐ Illumina	CRR453544
		Run‐079	Sample 3	Dataset‐3	sub‐dataset‐3‐ Illumina	CRR453548
		Run‐080	Sample 4	Dataset‐4	sub‐dataset‐4‐ Illumina	CRR453552
	Velvet	Run‐081	Sample 1	Dataset‐1	sub‐dataset‐1‐ Illumina	CRR453540
		Run‐082	Sample 2	Dataset‐2	sub‐dataset‐2‐ Illumina	CRR453544
		Run‐083	Sample 3	Dataset‐3	sub‐dataset‐3‐ Illumina	CRR453548
		Run‐084	Sample 4	Dataset‐4	sub‐dataset‐4‐ Illumina	CRR453552
	TIPPo	Run‐085	Sample 1	Dataset‐1	sub‐dataset‐1‐ Pacbio	CRR453538
		Run‐086	Sample 2	Dataset‐2	sub‐dataset‐2‐ Pacbio	CRR453541
		Run‐087	Sample 3	Dataset‐3	sub‐dataset‐3‐ Pacbio	CRR453545
		Run‐088	Sample 4	Dataset‐4	sub‐dataset‐4‐ Pacbio	CRR453549
	Oatk	Run‐089	Sample 1	Dataset‐1	sub‐dataset‐1‐ Pacbio	CRR453538
		Run‐090	Sample 2	Dataset‐2	sub‐dataset‐2‐ Pacbio	CRR453541
		Run‐091	Sample 3	Dataset‐3	sub‐dataset‐3‐ Pacbio	CRR453545
		Run‐092	Sample 4	Dataset‐4	sub‐dataset‐4‐ Pacbio	CRR453549
	NOVOPlasty	Run‐093	Sample 1	Dataset‐1	sub‐dataset‐1‐ Illumina	CRR453540
		Run‐094	Sample 2	Dataset‐2	sub‐dataset‐2‐ Illumina	CRR453544
		Run‐095	Sample 3	Dataset‐3	sub‐dataset‐3‐ Illumina	CRR453548
		Run‐096	Sample 4	Dataset‐4	sub‐dataset‐4‐ Illumina	CRR453552
	SPAdes	Run‐097	Sample 1	Dataset‐1	sub‐dataset‐1‐ Illumina sub‐dataset‐1‐ Pacbio	CRR453540 and CRR453538
		Run‐098	Sample 2	Dataset‐2	sub‐dataset‐2‐ Illumina sub‐dataset‐2‐ Pacbio	CRR453544 and CRR453541
		Run‐099	Sample 3	Dataset‐3	sub‐dataset‐3‐ Illumina sub‐dataset‐3‐ Pacbio	CRR453548 and CRR453545
		Run‐100	Sample 4	Dataset‐4	sub‐dataset‐4‐ Illumina sub‐dataset‐4‐ Pacbio	CRR453552 and CRR453549
	Unicycler	Run‐101	Sample 1	Dataset‐1	sub‐dataset‐1‐ Illumina sub‐dataset‐1‐ Pacbio	CRR453540 and CRR453538
		Run‐102	Sample 2	Dataset‐2	sub‐dataset‐2‐ Illumina sub‐dataset‐2‐ Pacbio	CRR453544 and CRR453541
		Run‐103	Sample 3	Dataset‐3	sub‐dataset‐3‐ Illumina sub‐dataset‐3‐ Pacbio	CRR453548 and CRR453545
		Run‐104	Sample 4	Dataset‐4	sub‐dataset‐4‐ Illumina sub‐dataset‐4‐ Pacbio	CRR453552 and CRR453549

### Metrics used to evaluate the performance of plant mitochondrial genome assemblers

The tools were evaluated from four dimensions: ease of setup, contiguity, completeness and correctness of their assembly results, as described previously (Freudenthal *et al*., 2020). Ease of installation was evaluated further on the basis of five metrics: possible automatic installation using conda or mamba, availability of installation instructions, availability of test data, requirement for particular installation and/or configuration, and requirement for particular database setup.

Contiguity reflected the extent to which a genome assembly comprised long, uninterrupted sequences or contigs, thereby indicating the assembly's ability to reconstruct large genomic regions without interruption. The assembly's contiguity was assessed by calculating the CC ratio, and the number of contigs was calculated using a custom python script, provided in Appendix S4.

Completeness was used to measure the genome assemblies and the gene contents. The assembly was mapped to the reference genome by using minimap2 to calculate the completeness score. In the resulting alignment, the percentage of bases of the reference genome aligned with bases from the assembly was defined as the coverage of reference (cov_ref). Conversely, the reference genome was mapped to the assembly by using minimap2. In the resulting alignment, the percentage of bases of the assembly aligned with bases from the reference was defined as the coverage of query (cov_qry).

Repeat resolution refers to the assemblers' ability to assemble the genome's repetitive sequences. The coverage between the assembled genome and the reference genome was compared in both directions to quantitatively evaluate repeat resolution. Then, repeat resolution was quantified as the minimum ratio between cov_qry and cov_ref. This value approached 1 when repeat regions were completely assembled. The calculation is expressed as follows (Freudenthal *et al*., 2020):

$$\text{Repeat Resolution}=\text{min}\;\left\{\frac{{\mathrm{cov}}_{\mathrm{qry}}}{{\mathrm{cov}}_{\mathrm{ref}}},\frac{{\mathrm{cov}}_{\mathrm{ref}}}{{\mathrm{cov}}_{\mathrm{qry}}}\right\}.$$

The completeness score was calculated by combining the metrics of completeness, repeat resolution and contig count (Freudenthal *et al*., 2020). The formula used for calculating the completeness score is as follows (Freudenthal *et al*., 2020):

$$\begin{array}{l} \text{score}=\frac{1}{4}\;\cdot \;\left({\mathrm{cov}}_{\mathrm{ref}}+{\mathrm{cov}}_{\mathrm{qry}}+\min\left\{\frac{{\mathrm{cov}}_{\mathrm{qry}}}{{\mathrm{cov}}_{\mathrm{ref}}},\frac{{\mathrm{cov}}_{\mathrm{ref}}}{{\mathrm{cov}}_{\mathrm{qry}}}\right\}+\frac{1}{n_{\text{contigs}}}\right)\;\cdot \;100. \end{array}$$

By contrast, the completeness of gene contents measured the numbers of genes that were found in the assembly results compared with those found in the reference genomes. The assembled genomes were annotated using PMGA (Li *et al*., 2025), and the copy numbers of each gene were counted. The difference between the copy numbers of all genes found in the assembly results and those found in the reference genomes was calculated, resulting in a score called relative copy number difference score (RCD), as follows:

$$\mathrm{RCD}=\frac{\sum_{i=1}^{n}\left|\text{Observed Copy Number}\;{\text{of Gene}}_{i}-\text{Expected Copy Number}\;{\text{of Gene}}_{i}\right|}{\sum_{i=1}^{n}\text{Copy Number}\;{\text{of Gene}}_{i}},$$

where ‘*n*’ represents the number of genes in the genome, ‘*i*’ represents the index of a particular gene and an RCD value of 0 indicates that the gene contents from the assemblies and the reference genomes were identical. The higher the RCD scores, the larger the deviations of the gene content of a genome assembly than those of the reference genome.

Correctness refers to the accuracy and fidelity of the assembly results. Key dimensions of the correctness include base accuracy and structural integrity. The assembly's correctness was evaluated using the newly released clipping reveals assembly quality (CRAQ) tool (Li *et al*., 2023). This tool assessed the assembly's quality by identifying and analysing clip‐based errors, including small‐scale clip‐based regional errors (CREs) and large‐scale clip‐based structural errors (CSEs). The raw sequencing data were mapped to the assembly results to calculate the correctness score. Then, CRAQ was used to analyse the resulting bam files, identify the existing CREs and CSEs, and the Regional Assembly Quality Index (R‐AQI) was calculated. The R‐AQI scores were used to assess the correctness of the assembly.

### Evaluation results

#### Ease of setup

All the software tools tested could be installed using conda or mamba except for NOVOPlasty, which did not require any installation at all. GetOrganelle required a separate step to configure the internal database.

#### Contiguity of *A. thaliana* and *O. sativa* assemblies

**Figure 4:**
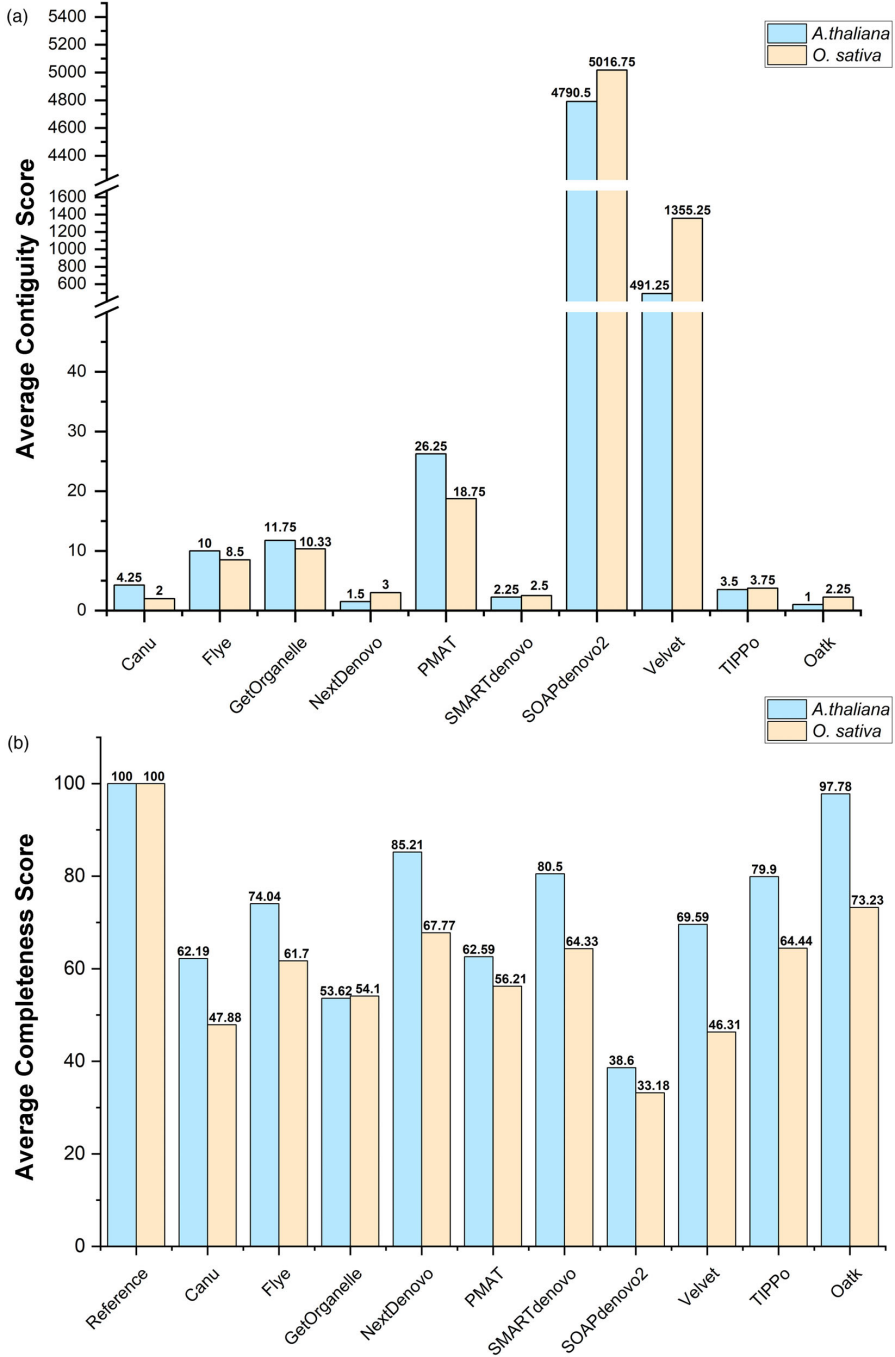
Number of contigs from different software tools used in the assembly of *A. thaliana* and *O. sativa* datasets. (a) Comparison of contiguity scores of mtDNA assemblies from *A. thaliana* (blue) and *O. sativa* (yellow) datasets by using various assembly software tools. The *x*‐axis categorizes the software and dataset types, and the *y*‐axis quantifies the contigs. A broken axis visualization technique is employed to effectively display smaller and larger values. (b) Comparison of completeness scores of mtDNA assemblies from *A. thaliana* (blue) and *O. sativa* (yellow) datasets by using various assembly software tools. The blue bars represent the integrity scores of *A. thaliana* mtDNA assemblies, plotted on the primary *y*‐axis. The orange bars graph represents the integrity scores of *O. sativa* mtDNA assemblies, plotted on the secondary *y*‐axis to the right. Each *x*‐axis category corresponds to a different software tool and dataset number.

The test dataset was assembled using different software tools with or without the enrichment of the mitochondrial reads. The enrichment procedure of the mitochondrial reads is described in section Selection criteria for plant mitochondrial genome assembly tools. The statistics of the assemblies obtained from the unenriched data are shown in Table S5, and the statistics of the assemblies obtained from the enriched data are shown in Table S6. The contiguity scores of various assembly results generated from the enriched data are shown in Figure 4a.

The reference genomes of the two species used in the test consisted of a single chromosome. As a result, the contig number and CC ratio of the assembly results are directly correlated. Among all tools tested, Oatk produced the fewest contigs, with only 1 contig for *A. thaliana* and 2.25 for *O. sativa*. NextDenovo performed well, producing 1.5 contigs for *A. thaliana* and 3 contigs for *O. sativa*. SMARTdenovo produced 2.25 contigs for *A. thaliana* and 2.5 for *O. sativa*.

#### Completeness of *A. thaliana* and *O. sativa* assembly results

##### Completeness of genome assembly

The completeness scores of the assemblies produced by different software tools are shown in Table S7. The highest possible score was 100, set by the scores obtained for the reference genomes of *A. thaliana* and *O. sativa* (Figure 4b). For *A. thaliana*, Oatk stood out as the top performer, achieving a score of 97.78. NextDenovo performed well, scoring 85.21. Similarly, SMARTdenovo performed well with a score of 80.5. For *O. sativa*, Oatk performed best, with a score of 73.23. SMARTdenovo and NextDenovo scored 67.77 and 64.33, respectively, ranking as the second and third best tools. Overall, Oatk, NextDenovo and SMARTdenovo emerged as the top‐performing tools, with Oatk performing particularly well with *A. thaliana* data, whereas NextDenovo demonstrated good results with both species.

##### Completeness of gene content

The RCD analysis results are shown in Table S8. The RCD scores of different assembly results showed a significant difference. For *A. thaliana*, the assemblies obtained from using Flye to assemble the dataset of sample 3 (in short Flye_sample_3), Flye_sample_4, GetOrganelle_sample_3, PMAT_sample_4 and Oatk_sample_4 showed RCD values close to zero. By contrast, the assemblies from Canu_sample_2 and SOAPdenovo2_samples_1–3 showed considerably large RCD values, reflecting substantial variations between the assemblies and the reference genome in terms of types of genes and their copy numbers.

Similarly, significant differences were observed in the completeness of gene contents among *O. sativa* assemblies obtained from different software tools (Table S9). The assembly results of Flye_sample_4, GetOrganelle_sample_4 and Flye_sample_3 exhibited RCD values close to zero. By contrast, Canu_sample_1 and Canu_sample_4 showed the highest RCD values, reflecting a substantial difference in the gene contents of the assemblies and those of the reference genomes.

In summary, the assemblies generated by tools, such as PMAT, GetOrganelle, Flye and Oatk, consistently showed high concordance with the reference genome.

#### Correctness of *A. thaliana* and *O. sativa* assemblies

**Figure 5:**
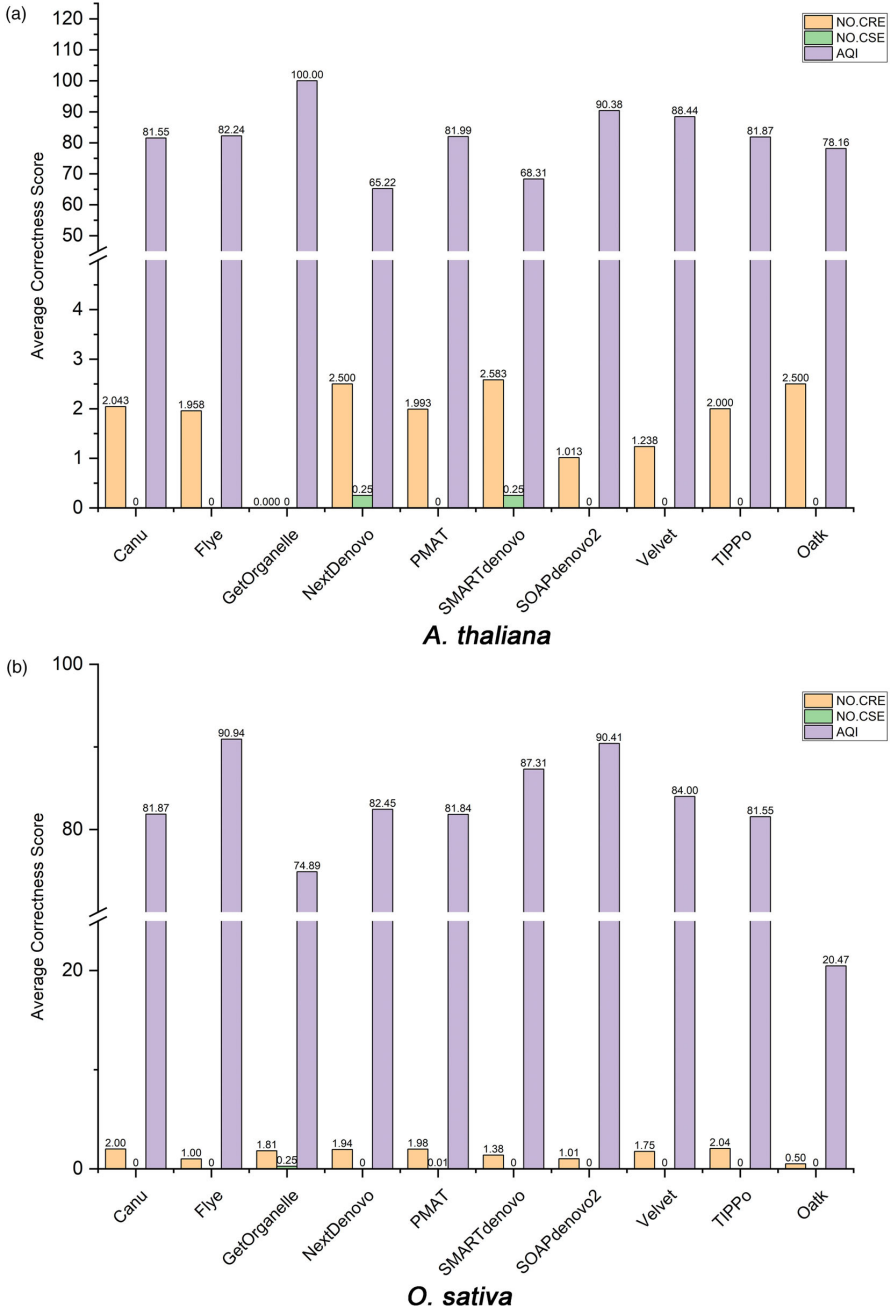
Evaluation of genome assembly correctness across various assemblers using CRAQ. Correctness is assessed using the CRAQ tool, which evaluates assembly fidelity by detecting clip‐based errors in mapped sequencing reads. Each assembler is evaluated on the basis of the number of CREs (orange bars) and CSEs (green bars) and the R‐AQI score (purple bars). (a) Correctness score of *A. thaliana*. (b) Correctness score of *O. sativa*.

The accuracy scores of the assemblies of *A. thaliana* and *O. sativa* are summarized in Figure 5 and Table S10. R‐AQI was used to assess the correctness of the assemblies, with the highest possible score being 100. Among the tested software tools, GetOrganelle stood out for both species, consistently achieving the highest R‐AQI scores of 100 across all samples. These results indicated that GetOrganelle produced highly accurate and error‐free assemblies for *A. thaliana* and *O. sativa*, with no detectable CREs or CSEs.

For *A. thaliana*, SOAPdenovo2 showed exceptional performance, obtaining R‐AQI scores consistently above 90 for its assembly results, ranging from 90.23 to 90.48 across different samples. These results reflected its ability to produce accurate mitochondrial genome assemblies with minimal errors. Similarly, the assembly results of Velvet showed slightly lower but still high enough R‐AQI scores, consistently exceeding 88.

For *O. sativa*, SOAPdenovo2 and Velvet were among the top performers. The assembly results of SOAPdenovo2 had R‐AQI scores ranging from 90.34 to 90.47. The assembly results of Velvet had similar R‐AQI scores, ranging from 90.36 to 90.43, highlighting its robustness in producing accurate assemblies across all samples. The assembly results of PMAT had R‐AQI scores ranging from 81.73 to 84.16, indicating solid assembly accuracy with relatively low error rates.

These results demonstrated that short reads combined with assemblers, such as GetOrganelle, SOAPdenovo2 and Velvet, are effective in generating accurate mitochondrial genome assemblies with minimal errors.

## Challenging issues in plant mitochondrial genome assembly and recommended solutions

### mtDNA enrichment

Plant cells contain three types of DNAs: nuDNAs, mtDNAs and ptDNAs. The mtDNAs accounted for only 2–3% of total DNAs (Makarenko *et al*., 2021). Therefore, close to 97–98% of total DNA is not useful for plant mitochondrial genome assembly. Several experimental strategies have been employed to enrich mtDNAs before downstream sequencing to overcome the above problems. First, density gradient centrifugation can be used to separate nuDNAs, mtDNAs and ptDNAs directly (Weber‐Lotfi *et al*., 2023). Second, antibodies targeting mitochondrial outer membrane proteins can be used to isolate the mitochondria for mtDNA extraction (Luo *et al*., 2020, 2021). Third, primers specific for mtDNAs could be designed to amplify mtDNAs (Cui *et al*., 2013). Lastly, custom‐designed probes could be used to enrich mtDNAs by hybridization (Sevigny *et al*., 2021).

Despite the numerous methods reported above, enriching mtDNA from total cellular DNA remains challenging. If mtDNAs are not enriched satisfactorily, bioinformatics methods can be used to enrich mtDNA reads. For example, reads of mtDNAs can be extracted from those of total DNAs on the basis of sequence similarity to a reference genome (Jin *et al*., 2020). Alternatively, reads of mtDNAs can be extracted from those of total DNAs on the basis of coverage depth to a set of pre‐assembled contigs. In this case, the reads are assembled using the de novo assembly algorithm. The reads are mapped to the pre‐assembled contigs, and the coverage depth is calculated. Those contigs having high coverage depth are more likely from ptDNAs. By contrast, those contigs having low coverage depth are more likely from nuDNAs. The contigs from mtDNAs can be obtained after contigs with too high or too low coverage depth are removed.

However, both bioinformatics enrichment methods have limitations. For the reference genome‐based method, finding the best reference plant mitochondrial genome that can retrieve all reads for the plant mitochondrial genome to be assembled is difficult because plant mitochondrial genomes are known to have a low level of conservation at their structure and the intergenic sequences. In addition, several factors can complicate the enrichment process on the basis of coverage depth. First, the library construction and DNA sequencing steps may create bias in the coverage depth for the regions sequenced. Second, repetitive sequences in mtDNAs may have higher coverage depth and be mistakenly considered to have originated from ptDNAs and removed. Third, sequences showing a high level of similarity among the three genomes may have high coverage depth and be removed mistakenly.

### Interference of horizontally transferred DNA sequences in the assembly process

Intracellular DNA exchanges have been well documented. MTPTs are mtDNA fragments that may have been transferred from the plastid genomes (Park *et al*., 2020; Warren and Sloan, 2020). Meanwhile, NUMTs are nuclear DNA fragments that may have been transferred from the mitochondrial genomes (Puertas and González‐Sánchez, 2020). mtDNA sequences may be similar to NUMT because of their common origin. MTPT sequences may be similar to some ptDNA sequences. NUMT or ptDNA sequences similar to MTPT sequences may be mistakenly identified as mtDNA sequences due to the high level of sequence similarity (Park *et al*., 2020; Tang *et al*., 2024), leading to incorrect plant mitochondrial genome assemblies (Jiang *et al*., 2023; Liu *et al*., 2023; Yang *et al*., 2022).

### High level of heterozygosity assembly

**Figure 6:**
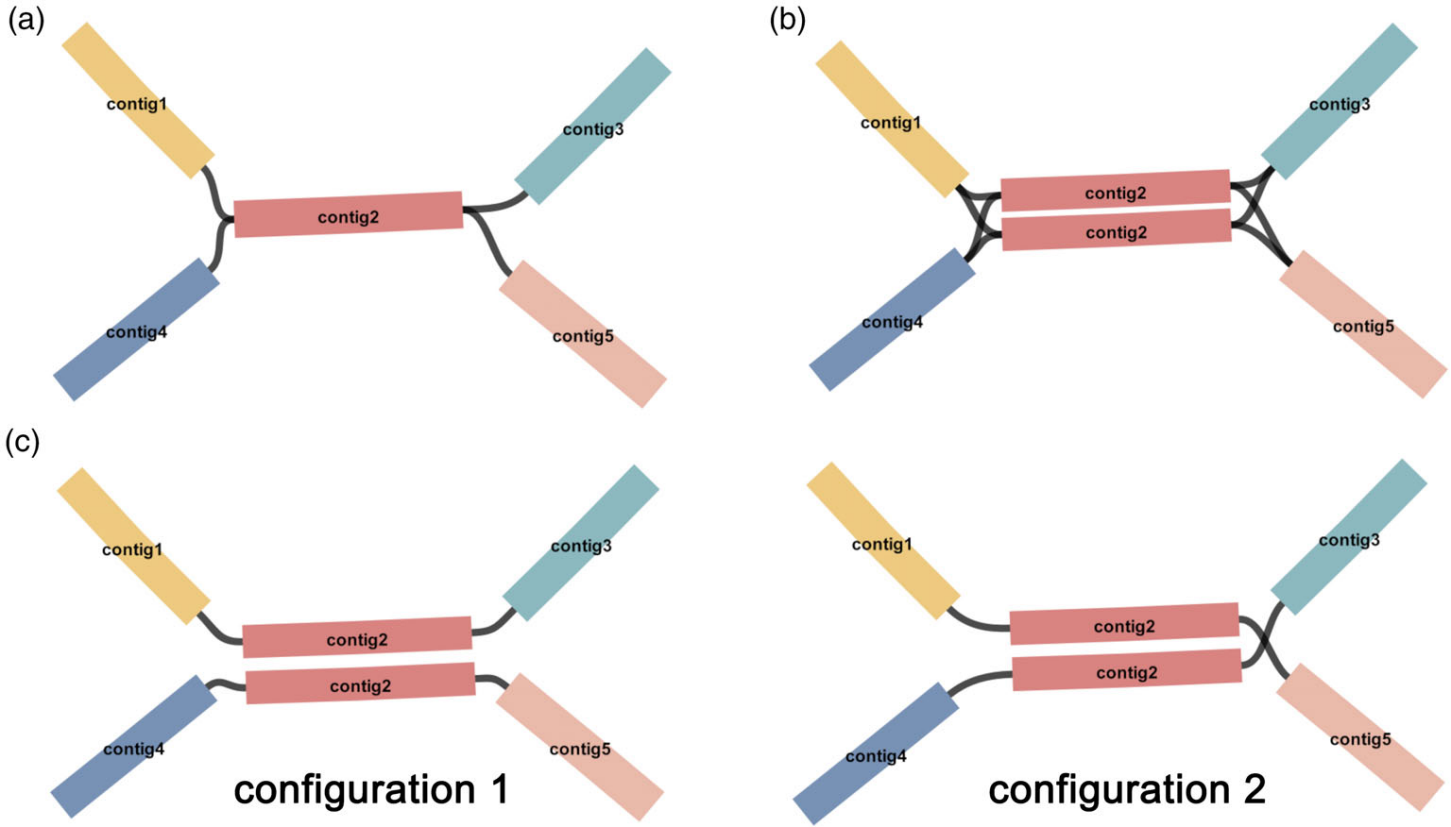
Schematic of the process to resolve a double‐bifurcation structure frequently found in plant mitochondrial genome assembly graphs. (a) Double‐bifurcating structure in the assembly graph. The black arrows show the paths to connect these contigs. (b) Simplified structures corresponding to the paths selected in (a), leading to two sequences: contig1‐contig5‐contig2 and contig3‐contig5‐contig4. (c) Paths alternative to those shown in (a). (d) Simplified structures corresponding to the path selected in (c), leading to two sequences: contig1‐contig5‐contig4 and contig3‐contig5‐contig2.

Heteroplasmy in plant mitochondrial genomes refers to the presence of multiple distinct mtDNA sequences within the cells of the same plant individual. Compared with nuDNAs, mtDNAs often exhibit frequent genome rearrangements resulting from recombination mediated by repetitive sequences (Browning and Browning, 2011; Kong *et al*., 2023). The long repetitive sequences lead to a large number of double‐bifurcating structures, which may represent the different plant mitochondrial genome configurations before and after the recombination (Figure 6) (Li *et al*., 2021; Shedge *et al*., 2007; Yang *et al*., 2023). How to verify the actual presence of these multiple plant mitochondrial genome configurations is a challenging problem.

### Difficulty in evaluating the quality of plant mitochondrial genome assemblies

Previous studies have recommended the usage of 3C properties, namely ‘Contiguity’, ‘Completeness’ and ‘Correctness’, to evaluate the quality of the plant mitochondrial genome assemblies (Li *et al*., 2023; Wang and Wang, 2023). ‘Comprehensiveness’ should be considered as another important property to evaluate the quality of a plant mitochondrial genome assembly.

#### Assessment of the contiguity of plant mitochondrial genome assembly

Three metrics were utilized to evaluate the contiguity of the plant mitochondrial genome assembly: the number of contigs, the contig N50 and the CC ratio. The number of contigs is instrumental in calculating the ratio of contigs to mitochondrial chromosomes, that is, CC ratio. A lower CC ratio indicated a higher assembly contiguity. A plant mitochondrial genome assembly without gaps yields a CC ratio of 1. Contiguity can be assessed using the N50 value as the length of the contig, the addition of which could cause the cumulative length of sorted contigs to exceed 50% of the length of all contigs. However, the N50 values could be affected by the exclusion of shorter contigs during the assembly process, making it less accurate. Therefore, the CC ratio is often considered a more reliable indicator of the contiguity of a plant mitochondrial genome assembly than N50.

#### Assessment of the completeness of plant mitochondrial genome assembly

The completeness of nuclear genome assemblies is usually assessed using Universal Single‐Copy Orthologues. A similar approach can be applied to evaluate plant mitochondrial genome assemblies (Mapleson *et al*., 2017). While previous studies have consistently shown that most plant mitochondrial genomes contain 24 conserved or core protein‐coding genes, recent research has revealed that the loss of these core genes in certain non‐parasitic lineages is not uncommon. For instance, studies by Mower (2020) and Butenko *et al*. (2024) suggest that the reasons different lineages retain different genes may be linked to their specific ecological and evolutionary histories. Therefore, the presence and number of these 24 core genes remain a crucial benchmark for evaluating the completeness of plant mitochondrial genome assemblies, but the potential gene loss in specific lineages should be considered. The variability in the number of other genes, including 17 variable protein‐coding genes and tRNA and rRNA genes among different species, highlights the diversity of mitochondrial genomes (Warren *et al*., 2021). The absence of some core genes in plant mitochondrial genome assemblies no longer solely indicates incompleteness but may reflect unique evolutionary traits of the species (Wu *et al*., 2022). This consideration should be adequately addressed when designing mitochondrial genome assembly and evaluation strategies.

For nuclear genome assemblies, telomeric and sub‐telomeric satellite arrays, centromeric satellite arrays, and ribosomal DNA loci are additional indicators of the genomes' completeness. This approach cannot be applied to plant mitochondrial genome assemblies at present because whether or not these repetitive sequences are present at the end of linear plant mitochondrial genome chromosomes is unknown.

For plastid genomes, the assembly's circularization is an important indicator of its completeness. However, this indicator cannot be used for plant mitochondrial genome assemblies containing linear chromosomes, which have been found present in many plant mitochondrial genome assemblies (Table S1).

#### Assessment of the correctness of plant mitochondrial genome assembly

Several methods have been developed to assess the correctness of a genome assembly at the base and structural levels. CRAQ calculates the scores of CREs and CSEs as an indicator of the plant mitochondrial genome's correctness at the single base or structural level.

#### Assessment of the comprehensiveness of plant mitochondrial genome assembly

Comprehensiveness is defined as the inclusion of all plant mitochondrial genome configurations. Previous studies have shown that plant mitochondrial genomes can have multiple configurations generated by repeat‐mediated recombination (Dong *et al*., 2018; Han *et al*., 2022; Jiang *et al*., 2023; Li *et al*., 2020, 2021; Liu *et al*., 2022; Wang and Wang, 2023). As a result, a high‐quality plant mitochondrial genome assembly should not only show a consensus plant mitochondrial genome sequence but also include various configurations. For simple plant mitochondrial genomes in *A. thaliana*, recent studies on the pan‐mitochondrial genome have explored the different structural configurations within them. Further research is still needed on other species and their plant mitochondrial genome configurations (Zou *et al*., 2025).

## General practice for assembling a plant mitochondrial genome

**Figure 7:**
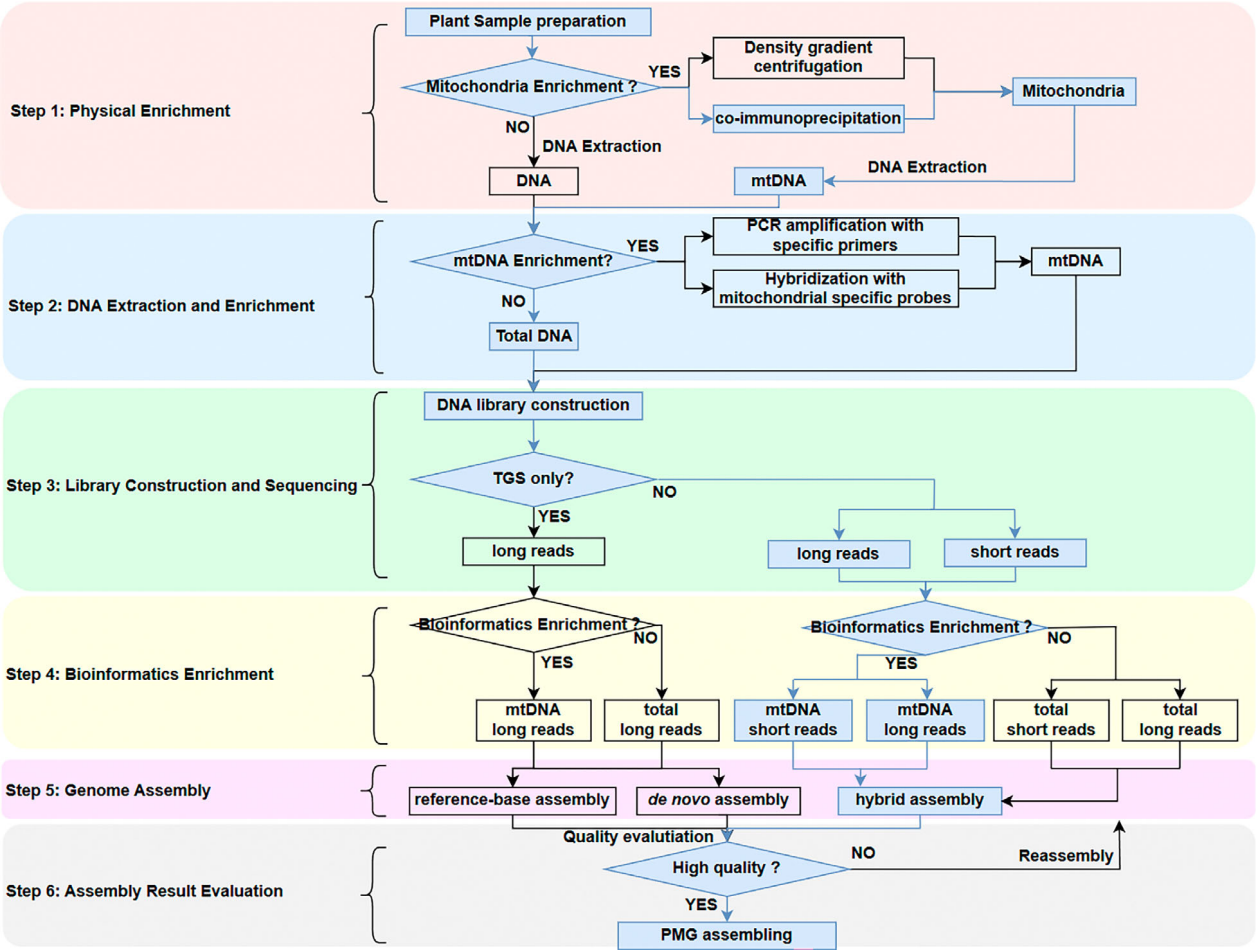
Proposed workflow for the entire process of assembling plant mitochondrial genomes, including enrichment. Sequencing and Assembling of mtDNAs, and Final Evaluation of Plant Mitochondrial Genome Assembly. The flow chart can be divided into six main steps. Initially, plant samples are either enriched for the mitochondria through density gradient centrifugation or targeted antibodies or proceed directly to DNA extraction. Then, the DNA is extracted and may be further enriched for mtDNA by using custom probes or specific primers. The libraries for long‐read data only or long‐ and short‐read data are constructed. Following sequencing, bioinformatics methods are used to enrich mtDNA reads from those of total DNA. The mtDNA sequences are assembled using reference‐based, *de novo*, or hybrid methods, and the genome assembly quality is evaluated. A reassembly may be needed if the assembly quality does not meet the requirement. The recommended process for plant mitochondrial genome assembly is shown in blue.

In the past 2 years, the authors' groups have sequenced, assembled, annotated, characterized and published 24 plant mitochondrial genomes, as listed in Note S1. A procedure that is most suitable for assembling the complete plant mitochondrial genome with currently available sequencing technologies and bioinformatics tools is summarized on the basis of experience gained through these studies (Figure 7).

### Sample preparation

Sample preparation should follow the instructions of the sequencing providers. In general, young and disease‐free plant tissues should be used for the extraction of DNAs to minimize damage and contamination of the exogenous DNAs. The tissues should be frozen immediately at −80 °C or stored in liquid nitrogen to prevent DNA degradation. If transportation is required, the samples should be placed on dry ice.

### Enrichment of mtDNA experimentally whenever possible

Enriching the mtDNAs whenever possible before proceeding with library construction and sequencing is strongly encouraged. Section Selection criteria for plant mitochondrial genome assembly tools introduces four experimental protocols for the enrichment of mtDNA prior to sequencing. However, the determination of the most appropriate method for a given species remains elusive. If possible, testing all these methodologies may be necessary to identify the optimal strategy.

### DNA extraction and sequencing library preparation

This step should be performed following the procedure recommended by the manufacturers.

### Using bioinformatics methods to enrich reads

Enriching mitochondrial reads through bioinformatics methods aims to reduce interference of those reads from nuDNAs and ptDNAs. In the presence of a reference plant mitochondrial genome, one can map all sequence reads to it. The mapped reads are considered to be mitochondrial reads and should be selected for the downstream assembly process. The suggested protocol could be found in Note S6.

Alternatively, the mitochondrial reads can be enriched on the basis of k‐mer frequency distribution. The ptDNA reads can be removed using reference sequences. For the remaining reads, the frequency distribution of all k‐mers in the sequencing data can be calculated. Thus, high‐frequency k‐mers can be identified. These high‐frequency k‐mers are typically associated with high‐copy‐number genomes. In mtDNA read enrichment, high‐frequency k‐mers are most likely derived from mtDNA sequences, whereas low frequency or randomly distributed k‐mers are more often linked to nuDNAs. These candidate k‐mers are then employed to filter the sequencing reads, isolating those that contain the selected k‐mers. The resulting subset of reads is retained as putative mtDNA sequences for further analysis (Li *et al*., 2019). The suggested protocol could be found in Note S7.

### Assembling plant mitochondrial genome

Using the de novo method is recommended for plant mitochondrial genome assembly, as detailed in subsection Overview. Two methods using short‐ and long‐read data were discussed. Based on the authors' experience, method 1 is more effective. Initially, these enriched mitochondrial short‐read data are assembled de novo to generate a draft assembly. The long‐read data can then be mapped to this draft assembly, and then those mapped long‐read data are selected. Finally, Unicycler is employed to assemble the plant mitochondrial genome by using the original short‐read data and the selected long‐read data. The suggested protocol could be found in Note S8.

### Assessment of assembly quality

The quality of the assembled genome can be assessed through the following steps: First, circularity is used as an indicator of the completeness of an assembly because the majority of plant mitochondrial genomes are circular. Tools, such as Circlator (Hunt *et al*., 2015), can be employed to verify the closure of circular mitochondrial chromosomes. This method cannot be applied to plant mitochondrial genomes containing linear mitochondrial chromosomes. Second, the completeness of the plant mitochondrial genome can be assessed on the basis of its gene contents. The assembled genome should be annotated using tools, such as PMGA, to confirm the presence of 41 coding sequences, with a particular focus on the 24 core genes (Li *et al*., 2025). Third, the depth of coverage can be used. A coverage depth of 20x was used as a threshold to determine if a region is covered sufficiently (Jackman *et al*., 2020). Lastly, the long‐read data can be mapped to the assembly to determine whether or not a sequence is misassembled. The presence of long‐read data that can span the repetitive regions could indicate the correct assembly of those regions.

### Short protocol for PMG assembly

As described above, PMG analysis is still under rapid development, and no cross‐taxa unified workflow is available at this moment. However, for those who plan to carry out this analysis, the following procedure is recommended: First, the mitochondria should be isolated, and the mtDNA should be extracted. Then, long‐read sequencing of mtDNA could be performed using either the Nanopore R10 or PacBio HiFi platform, while NGS sequencing could be carried out using the Illumina NovaSeq 6000 platform. Although the PacBio HiFi platform offers accuracy reads, our benchmarking results (section Correctness of *A. thaliana* and *O. sativa* assemblies) indicate that assemblies using Illumina reads exhibit superior performance with respect to the correctness metric. Next, the ‘de novo‐short/long method 3’ (section De novo assembly algorithm using short‐ and long‐read data) could be used for hybrid assembly (Figure 2b). The details are as follows: Tools like NextDenovo could be used to perform de novo assembly of long reads. The BLASTn tool should be used to compare the mitochondrial protein‐coding gene sequences and the assembled contigs. The Seqkit tool could be used to extract contigs matching those mitochondrial genes. The short and long reads can be individually aligned to the selected contigs by using BWA, and the mapped reads can be extracted using SAMtools. Subsequently, the filtered short and long reads can be subjected to hybrid assembly with Unicycler to generate the final genome assembly.

## Conclusions

Here, the challenges in assembling plant mitochondrial genomes were reviewed. The three main mitochondrial genome assembly algorithms, RB, de novo and IME, were discussed, outlining their advantages and disadvantages. The most used assemblers were reviewed and discussed on the basis of the types of input data they accept and the algorithms they employ. While each tool exhibits specific strengths and weaknesses, SMARTdenovo, NextDenovo, Oatk, PMAT and GetOrganelle performed the best overall, based on the metrics of continuity, completeness and accuracy. Among them, Oatk performed well in terms of continuity and completeness. However, it could process high‐accuracy reads only, such as PacBio HiFi data. SMARTdenovo and NextDenovo showed continuity scores, second only to Oatk, and they could handle data from different platforms. However, they failed to generate assembly graphs. PMAT exhibited good gene completeness, but it currently does not analyse assembly graphs. GetOrganelle achieved the best accuracy, but with NGS data only. Lastly, a general practice is proposed, and a short protocol for plant mitochondrial genome assembly is recommended.

## Competing interests

The authors declare that they have no competing or conflicting interests.

## Funding

The study was supported by the National Natural Science Foundation of China (81872966), the CAMS Innovation Fund for Medical Sciences (CIFMS) (2021I2M‐1‐022), the National Science & Technology Fundamental Resources Investigation Program of China (2024FY100700), Hunan Provincial Special Project for Construction of Innovation Demonstration Area at Chenzhou City under National Sustainable Development Plan (2023sfq04) and the Guangxi Laibin City Science Research and Technology Development Plan (202410). These funders were neither involved in the study design, data collection, analysis, publication decision, nor manuscript preparation.

## Author contributions

CL conceived the study, YN and CL wrote the manuscript, YHT drew the figures, and CL and JLL reviewed the manuscript critically. All authors read and approved the manuscript.

## Supporting information


**Figure S1** Schematic representation of the PMG assembling algorithms.
**Figure S2** Draft assembly of *A. thaliana* mitochondrial genome using short reads.
**Figure S3** Resolving the graph into a major conformation of the mitogenome of *A. thaliana*.

**Table S1** Summary of published plant mitochondrial genomes, their structures and the assembly methods.
**Table S2** Number of studies using particular types of DNA sequencing technologies in plant mitochondrial genome studies from 1992 to 2024.
**Table S3** Number of studies using particular algorithms to assemble mitochondrial genomes over time.
**Table S4** Number of studies using particular software tools to assemble the mitochondrial genomes during the past twenty years.
**Table S5** Statistics of the genome assembly results of two test species by using different software tools.
**Table S6** Contiguity of the mitochondrial genome assemblies for the two species assembled by using different software tools.
**Table S7** Completeness of the genome assemblies for the two species assembled by using different software tools.
**Table S8** Completeness of the gene contents found in the *A. thaliana* genome assemblies resulting from different softwares tools.
**Table S9** Completeness of the gene contents found in the *O. sativa* genome assemblies resulting from different softwares tools.
**Table S10** Correctness of the mitochondrial genome assembly for the test datasets.

**Note S1** List of papers related to plant mitochondrial genomes from our group in the past years.
**Note S2** Additional information for the complexity of PMG Assembly.
**Note S3** Additional assemblers for PMG.
**Note S4** Computational environment and server hardware configuration for experimental tools setup.
**Note S5** Method to extract mitochondrial contigs from the assembly results of whole genome sequencing data.
**Note S6** The protocol to enrich mtDNA reads based on their sequence similarity to the reference genome.
**Note S7** The protocol to enrich mtDNA reads based on kmer distribution.
**Note S8** The recommended protocol to assemble a PMG.

**Appendix S1** The config file for the NOVOPlasty software.

**Appendix S2** The config file for the Nextdenovo software.

**Appendix S3** The script for filtering contigs based on alignment to a mitochondrial reference genome.

**Appendix S4** The script for calculating basic assembly statistics from FASTA files.

**Appendix S5** The script for evaluating assembly completeness using the formula.

## Data Availability

The data that supports the findings of this study are available in the supplementary material of this article.
