# Supplementary material for: Advance in the assembly of the plant mitochondrial genomes using high‐throughput DNA sequencing data of total cellular DNAs
Source: Plant Biotechnol J. 2025 Jul 29;23(11):4944–65. doi: 10.1111/pbi.70249 (PMC12576441; doi:10.1111/pbi.70249)
Supplement: Supplementary file 1 — Figure S1 Schematic representation of the PMG assembling algorithms. Figure S2 Draft assembly of A. thaliana mitochondrial genome using short reads. Figure S3 Resolving the graph into a major conformation of the mitogenome of A. thaliana. [file PBI-23-4944-s003.docx]

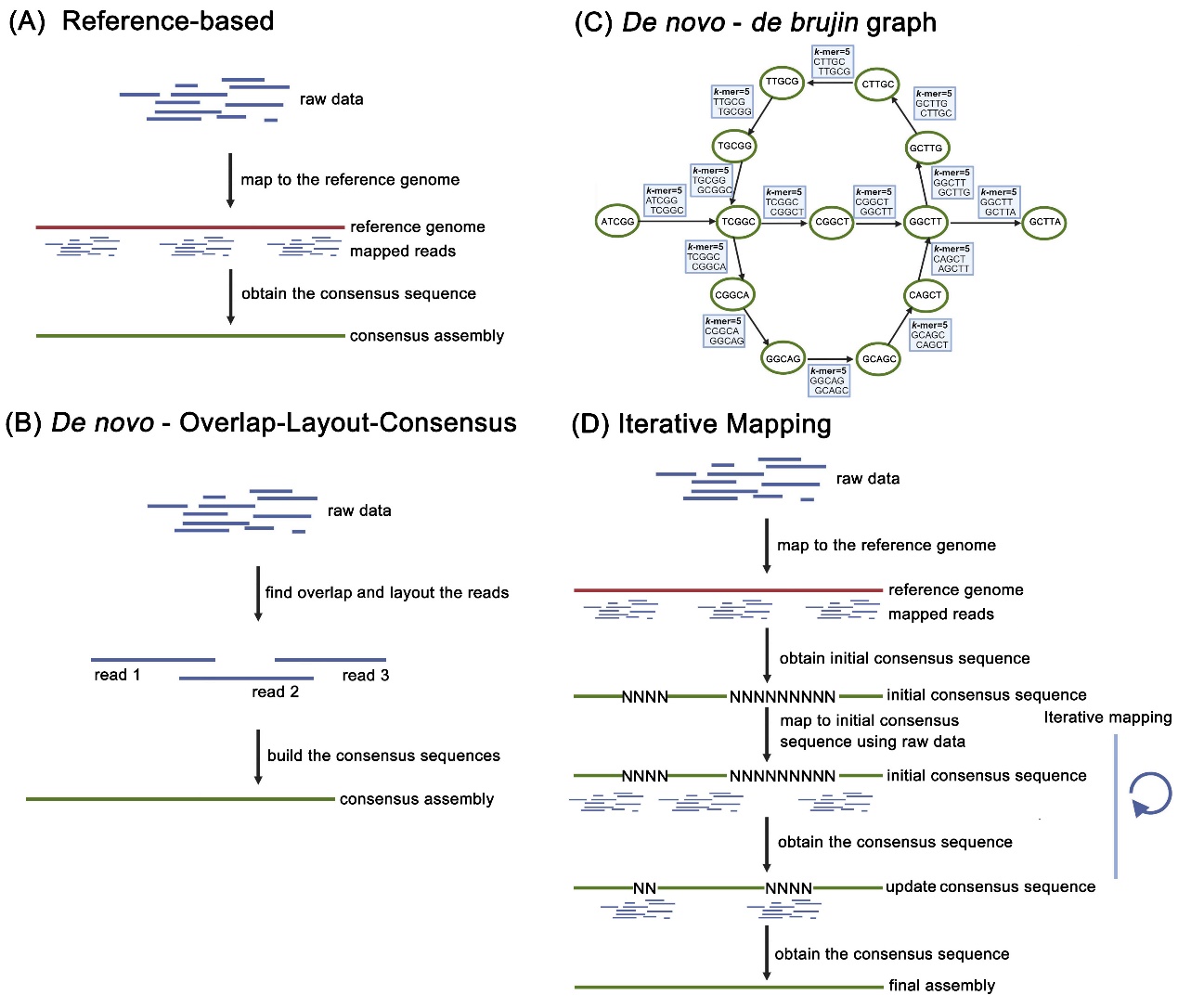


**Figure S1 Schematic representation of the PMG assembling algorithms. (A)** Reference-based assembly: Sequencing reads are firstly mapped to a reference genome. Those mapped reads were subject to De novo assembly to obtain contigs. **(B) De novo assembly: Overlap-layout-consensus (OLC)**. This algorithm with the assembly of contigs from sequencing reads based on the overlapping of the end of the sequence reads. Large-insert reads are used link contigs. Gaps are filled when possible, resulting in a genome assembly. **(C)** **De novo assembly:** De Bruijn graph assembly (k=5): A graphical representation of k-mers (k=5), where sequences overlap by k-1 nucleotides. A path is found to connect as many node as possible. **(D) Iterative mapping**: Sequencing reads are mapped to a seed sequence, and these reads are selected and assembled. The newly assembled seqences are placed at the end of the seed sequence to extend the seed sequence. This process is repeated until the assembly sequence can no longer be extended.

**
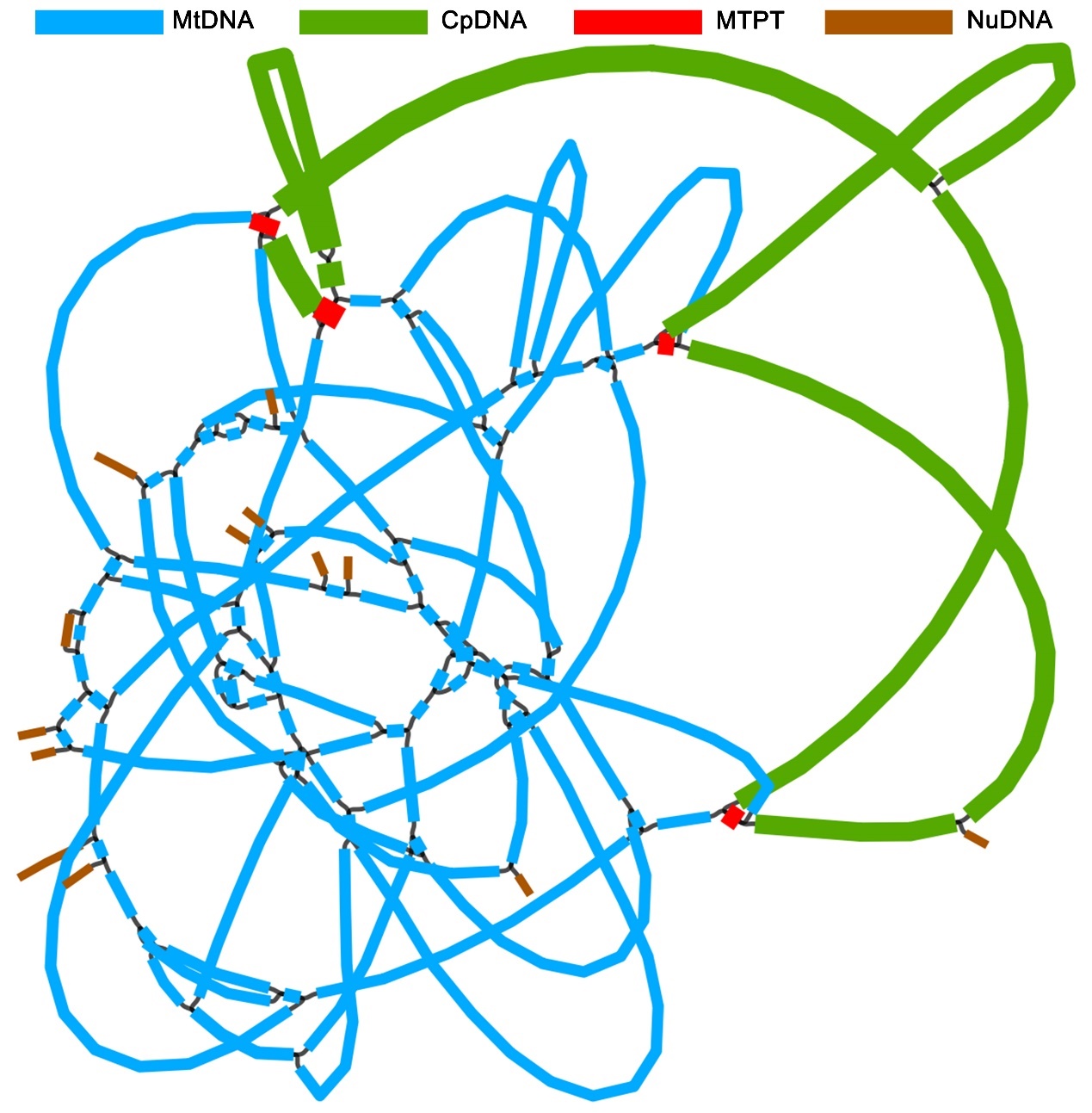
**

**Figure S2 Draft Assembly of *A. thaliana* Mitochondrial Genome Using Short Reads.** This figure illustrates the preliminary assembly results of the *A. thaliana* mitochondrial genome from short read sequences processed by using GetOrganelle with default parameters. In the graph, each colored thick lines represent a node. Edges among the nodes represent the connections of the contigs. Contigs derived from different sources, such as mtDNA, cpDNA, MTPT, nuDNA, are labeled with different colors as shown above the graph.


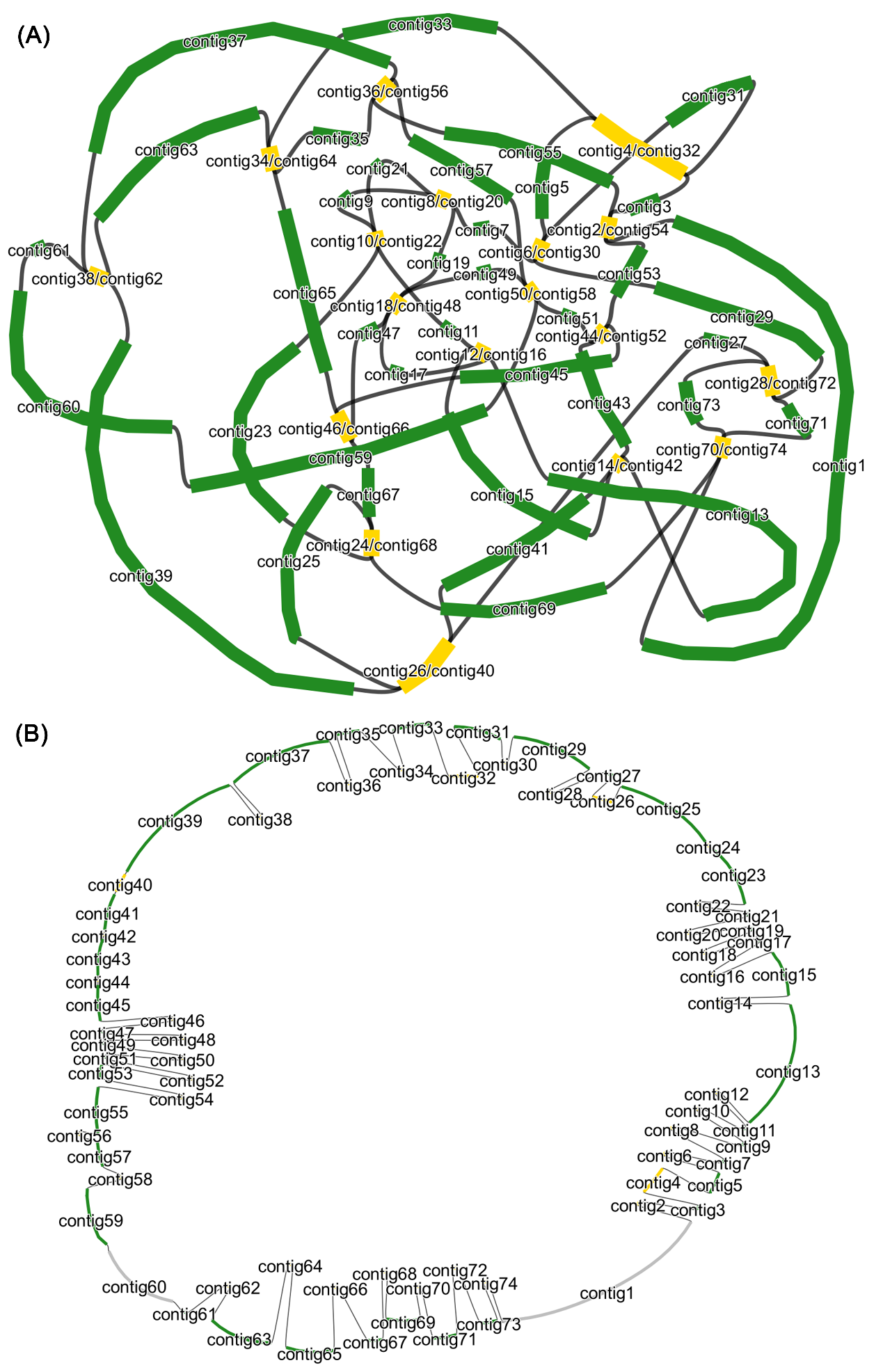


**Figure S3 Resolving the graph into a major conformation of the mitogenome of *A. thaliana*.** This figure presents the assembly of the *A. thaliana* mitochondrial genome. **(A)** displays the *de Bruijn* graph of short reads which represent the preliminary assembly structure of the genome, illustrating 74 contigs and their connections. **(B)** depicts the final circular assembly result resolved with long reads, showcasing how longer sequences aid in bridging gaps and resolving repetitive regions. The contigs in deep green denote the unique, single-copy regions of the mitochondrial genome, while the yellow regions highlight the repetitive sequences. The resolution of these repetitive areas is critical for accurate genome assembly and is effectively demonstrated in this figure.
