# Supplementary material for: Advance in the assembly of the plant mitochondrial genomes using high‐throughput DNA sequencing data of total cellular DNAs
Source: Plant Biotechnol J. 2025 Jul 29;23(11):4944–65. doi: 10.1111/pbi.70249 (PMC12576441; doi:10.1111/pbi.70249)
Supplement: Supplementary file 3 — Note S1 List of papers related to plant mitochondrial genomes from our group in the past years. Note S2 Additional information for the complexity of PMG Assembly. Note S3 Additional assemblers for PMG. Note S4 Computational environment and server hardware configuration for experimental tools setup. Note S5 Method to extract mitochondrial contigs from the assembly results of whole genome sequencing data. Note S6 The protocol to enrich mtDNA reads based on their sequence similarity to the reference genome. Note S7 The protocol to enrich mtDNA reads based on kmer distribution. Note S8 The recommended protocol to assemble a PMG. [file PBI-23-4944-s007.docx]

**Supplementary Note 1: List of Papers related to Plant Mitochondrial Genomes from Our Group in the Past Years**

| **No.** | **Title** | **Journal** | **Year** |
| --- | --- | --- | --- |
| 1 | PMGA: A Plant Mitochondrial Genome Annotator | Plant Communications | 2024 |
| 2 | Repeat-mediated recombination results in Complex DNA structure of the mitochondrial genome of *Trachelospermum jasminoides* | BMC Plant Biology | 2024 |
| 3 | Analysis of the complete mitochondrial genome of Panax quinquefolius reveals shifts from cis-splicing to trans-splicing of intron cox2i373 | Gene | 2024 |
| 4 | Comprehensive analysis of the mitochondrial genome of Rehmannia glutinosa: insights into repeat-mediated recombinations and RNA editing-induced stop codon acquisition | Frontiers in Plant Science | 2024 |
| 5 | Plant mitochondrial genome map (PMGmap): A software tool for the comprehensive visualization of coding, noncoding and genome features of plant mitochondrial genomes | Molecular Ecology Resources | 2024 |
| 6 | The Complete Mitochondrial Genome of Paeonia lactiflora Pall. (Saxifragales: Paeoniaceae): Evidence of Gene Transfer from Chloroplast to Mitochondrial Genome | Genes | 2024 |
| 7 | Genetic diversity of Coffea arabica L. mitochondrial genomes caused by repeat- mediated recombination and RNA editing | Frontiers in Plant Science | 2023 |
| 8 | Complete mitochondrial genome of Mentha spicata L. reveals multiple chromosomal configurations and RNA editing events | International Journal of Biological Macromolecules | 2023 |
| 9 | The mitochondrial genomes of Panax notoginseng reveal recombination mediated by repeats associated with DNA replication | International Journal of Biological Macromolecules | 2023 |
| 10 | Mitochondrial genome of Artemisia argyi L. suggested conserved mitochondrial protein-coding genes among genera Artemisia, Tanacetum and Chrysanthemum | Gene | 2023 |
| 11 | Mitochondrial Genome Sequence of Salvia officinalis (Lamiales: Lamiaceae) Suggests Diverse Genome Structures in Cogeneric Species and Finds the Stop Gain of Genes through RNA Editing Events | International Journal of Molecular Sciences | 2023 |
| 12 | Characterisation of the complete mitochondrial genome of Taraxacum mongolicum revealed five repeat-mediated recombinations | Plant Cell Reports | 2023 |
| 13 | Polymeric Structure of the Cannabis Sativa L. mitochondrial genome identified with an assembly graph model | Gene | 2023 |
| 14 | De Novo Hybrid Assembly of the Salvia miltiorrhiza Mitochondrial Genome Provides the First Evidence of the Multi-Chromosomal Mitochondrial DNA Structure of Salvia Species | International Journal of Molecular Sciences | 2023 |
| 15 | Cistanche Species Mitogenomes Suggest Diversity and Complexity in Lamiales-Order Mitogenomes | Genes | 2022 |
| 16 | Comparative analysis of the plastid and mitochondrial genomes of Artemisia giraldii Pamp | Scientific Reports | 2022 |
| 17 | Comparative analysis of the chloroplast and mitochondrial genomes of Saposhnikovia divaricata revealed the possible transfer of plastome repeat regions into the mitogenome | BMC Genomics | 2022 |
| 18 | Assembly of the complete mitochondrial genome of an endemic plant, Scutellaria tsinyunensis, revealed the existence of two conformations generated by a repeat-mediated recombination | Planta | 2021 |

**Supplementary Note 2: Additional information for the complexity of PMG Assembly**

The complexity of PMGs can be attributed to several critical factors, including frequent recombination and replication events, the integration of foreign genetic material, gene loss, and rearrangements. NGS technologies, such as Illumina, provide high-accuracy short-read sequencing, which can only detect minor genetic variations. However, short reads generated by NGS technologies cannot resolve the regions containing the repetitive sequences, such as those found in PMGs.

For example, analyses on the *Arabidopsis* *msh1* mutants using Pacbio HiFi data displayed structural variations, such as single nucleotide polymorphisms and indels in both mitochondrial and plastid genomes, providing a detailed mitochondrial genome recombination landscape.

**Supplementary Note 3: Additional Assemblers for PMG**

**1. MITObim**

MITObim utilized the IMEAA. MITObim used the MIRAbait tool to identify reads mapped to the bait/seed sequences, and then the MIRA software to assemble these mapped reads (Table 1). The map/assemble processes were repeated until no more new reads were found. MITObim has been widely used for animal mitochondrial genome assembly. In plants, it has only been used once in the past five years (Supplementary Table 1).

**2.** **SAGBAC**

SAGBAC utilized DNAA (DNAA-NGS-TGS, method2) to assemble both NGS and TGS data. NGS data were first assembled using CLC, IDBA, and MIRA software, and the results are integrated. The raw data were then aligned to these assembled contigs using BWA, and samtools was employed to calculate the coverage depth. Contigs with a coverage depth of 3000x or higher were retained. Subsequently, the Iterative Sequence Ends Identity Search (ISEIS) algorithm constructed a mitochondrial assembly graph based on the overlapping regions between contig sequences. The R package igraph was utilized for graph visualization. For long-read data, BLAST was used to sort the contigs. Then, connection paths between them were determined, followed by manually simplification of the mitochondrial graph to obtain a complete reference mitochondrial genome. This tool was semi-automated and required further development to become a fully automated, turn-key solution. SAGBAC has been cited for 1 time in the past five years (Table 2, Supplementary Table 1).

**3. MitoHiFi**

MitoHiFi utilized the RBAA to assemble PacBio HiFi sequencing data. Firstly, MitoHiFi mapped PacBio HiFi reads to the reference genome and used a specific length filtering strategy to exclude reads likely originating from nuclear mitochondrial DNA segments (NUMTs). Then, MitoHiFi used Hifiasm to assemble the filtered reads. MitoHiFi is particularly suitable for assembling PMGs having reference genomes.

**4. Organelle-PBA**

Organelle_PBA utilized the RBAA assemble PacBio reads in multiple formats. Initially, Organelle_PBA mapped PacBio reads to the reference genome using BlasR. Organelle_PBA selected mapped PacBio reads and assembled them using the Spray software. The size of the assembly was compared with that of the reference genome to evaluate the assembly quality. As RBAA assemblers, both Organelle_PBA and MitoHiFi’s performance depended on the selection and quality of reference genomes.

**5. GSAT**

GSAT utilized DNAA (DNAA-NGS-TGS, method2) to assemble both NGS and TGS data. Firstly, GSAT called the SPAdes software to construct an initial assembly graph using the NGS data. It then employed the GraphFilt algorithm to filter out contigs from the ntDNAs and ptDNAs based on read coverage depth. Subsequently, TGS data was used to refine the initial assembly graph to generate the master PMG assembly graph. GSAT have been cited for 1 time in the past five years (Table 2, Supplementary Table 1)

**Supplementary Note 4: Computational Environment and Server Hardware Configuration for Experimental Tools Setup**

All tools were executed in mamba environments on a single server featuring an Intel® Xeon® Silver 4316 CPU with 80 CPUs across 2 sockets, hyperthreading enabled, 2 threads per core, and 20 cores per socket. The server is equipped with 192 GB of RAM and 1 TB of storage, running CentOS Linux release 7.9.2009 (Core). This setup mirrors that found in typical research laboratories.

**Supplementary Note 5: Method to extract mitochondrial contigs from the assembly results of whole genome sequencing data**

All these tools were used assembly the data derived the whole genome without any filtering. These assembly results contained many contigs that correspond to those of the plastomes and nuclear genomes. As the purpose of this study is to evaluate their performance on the PMGs, we filtered the whole genome assembly with the reference genomes, resulting in the PMG assemblies. The performance evaluation was then conducted with the PMG assemblies. To isolate contigs originated from PMGs, the contigs assembled from unfiltered raw data were compared against reference genomes. Contigs exhibiting over 90% sequence similarity with regions of the reference genome were considered as putative mitochondrial genome fragments. The procedure was as follows: the assembly results were used as queries to search against a blast database constructed with the reference genome sequences by using BLASTn. The alignment lengths of multiple matches for a given contig were summed up, and if the cumulative length surpassed 90% of the contig's total length, the contig was retained as potential mitochondrial contigs . This analysis was carried out using a custom script provided in Appendix 3.

**Supplementary Note 6: The Protocol to Enrich mtDNA Reads Based on Their Sequence Similarity to the Reference Genome**

**Setup**

Reference genome: reference_genome.fa

Second-generation sequencing data: NGS_R1.fq and NGS_R2.fq

Third-generation sequencing data: long_reads.fq

Output prefix: filtered_reads

**Commands used for Enriching Second-Generation Sequencing Read**

(1) Align the reads to the reference genome using Minimap2 and generate a SAM file:
$ minimap2 -ax sr reference_genome.fa NGS_R1.fq NGS_R2.fq > filtered_reads.sam

(2) Convert the SAM file to a BAM file, filtering out unaligned reads using Samtools:
$ samtools view -@ 16 -bS -F 12 filtered_reads.sam > filtered_reads.bam

(3) Extract the filtered reads back into FASTQ format using Bedtools:
$ bedtools bamtofastq -i filtered_reads.bam -fq filter_R1_filtered_reads.fq -fq2 filter_R2_filtered_reads.fq

### **Commands used for Enriching Third-Generation Sequencing Read**

1. Align the reads to the reference genome using Minimap2 and generate a SAM file:
   $ minimap2 -a reference_genome.fa long_reads.fq > filtered_reads.sam
2. Convert the SAM file to a BAM file, filtering out unaligned reads using Samtools:
   $ samtools view -@ 16 -bS -F 12 filtered_reads.sam > filtered_reads.bam
3. Extract the filtered reads back into FASTQ format using Bedtools:
   $ bedtools bamtofastq -i filtered_reads.bam -fq filtered_reads.fq

(4) Remove duplicate reads from the FASTQ file using Seqkit:
$ seqkit rmdup -n -i filtered_reads.fq -o filtered_reads_rmdup.fq

**Supplementary Note 7: the protocol to enrich mtDNA reads based on kmer distribution**

**Setup**
Reference genome: reference_genome.fa
Second-generation sequencing data: NGS_R1.fq and NGS_R2.fq
Third-generation sequencing data: long_reads.fq
Kmer file: kmer_list.txt (a file containing the kmer sequences used for filtering)
Output prefix: kmer_enriched_reads

**Commands used for Enriching Second-Generation Sequencing Reads**

(1) Use Jellyfish to generate kmer counts from the reference genome:
$ jellyfish count -m 21 -s 100M -t 16 reference_genome.fa -o reference_genome.jf

(2) Extract the kmer sequences from the Jellyfish output:
$ jellyfish dump reference_genome.jf > kmer_list.txt

(3) Filter the reads based on the kmer sequences using Seqkit for Read 1:
$ seqkit grep -f kmer_list.txt -o filtered_R1_kmer_enriched_reads.fq NGS_R1.fq

(4) Filter the reads based on the kmer sequences using Seqkit for Read 2:
$ seqkit grep -f kmer_list.txt -o filtered_R2_kmer_enriched_reads.fq NGS_R2.fq

**Commands used for Enriching Third-Generation Sequencing Reads**

(1) Use Jellyfish to generate kmer counts from the reference genome:
$ jellyfish count -m 21 -s 100M -t 16 reference_genome.fa -o reference_genome.jf

(2) Extract the kmer sequences from the Jellyfish output:
$ jellyfish dump reference_genome.jf > kmer_list.txt

(3) Filter the reads based on the kmer sequences using Seqkit:
$ seqkit grep -f kmer_list.txt -o filtered_kmer_enriched_reads.fq long_reads.fq

(4) Remove duplicate reads from the filtered output using Seqkit:
$ seqkit rmdup -n -i filtered_kmer_enriched_reads.fq -o filtered_kmer_enriched_reads_rmdup.fq

**Supplementary Note 8: the recommended protocol to assemble a PMG**

**Setup**
Reference mitochondrial gene sequence: mitochondrial_gene.fa
Third-generation sequencing data: long_reads.fq
Second-generation sequencing data: NGS_R1.fq and NGS_R2.fq
Flye assembly output: flye_output/assembly.fasta
Output directory: output_dir

**Commands**

**Step 1: De novo assembly of TGS reads using Flye**
(1) Perform de novo assembly using Flye:
$ flye --nano-raw long_reads.fq --out-dir flye_output

**Step 2: Compare the contig sequences and the mitochondrial gene sequences using BLAST**
(1) Perform a BLAST search to identify assembly contigs containing the mitochondrial gene:
$ blastn -query mitochondrial_gene.fa -subject flye_output/assembly.fasta -outfmt 6 -out mitochondrial_blast_results.txt -evalue 1e-5

(2) Parse the BLAST results to extract matching sequence names:
The BLAST output in mitochondrial_blast_results.txt (in -outfmt 6 format) contains tab-delimited columns. Extract the first column (qseqid), which corresponds to matching sequence names:

$ awk ‘{print $2}’ mitochondrial_blast_results.txt | sort | uniq > matched_contigs_list.txt

(3) Use seqkit to extract the contigs from the Flye assembly file:
$ seqkit grep -f matched_contigs_list.txt flye_output/assembly.fasta -o mitochondrial_contigs.fasta

**Step 3: Map NGS reads to the mitochondrial contigs**
(1) Align NGS reads to the mitochondrial contigs using Minimap2:
$ minimap2 -ax sr mitochondrial_contigs.fasta NGS_R1.fq NGS_R2.fq > ngs_to_mitochondria.sam

(2) Convert the SAM file to BAM format and filter for aligned reads using Samtools:
$ samtools view -@ 16 -bS -F 12 ngs_to_mitochondria.sam > ngs_to_mitochondria.bam

(3) Extract the aligned reads into separate FASTQ files using Bedtools:
$ bedtools bamtofastq -i ngs_to_mitochondria.bam -fq short_reads_1.fastq.gz -fq2 short_reads_2.fastq.gz

**Step 4: Assemble PMGs using Unicycler with hybrid data**
(1) Perform hybrid assembly with Unicycler, combining filtered NGS reads and all TGS reads:
$ unicycler -1 short_reads_1.fastq.gz -2 short_reads_2.fastq.gz -l long_reads.fq -o output_dir
